# Multi spectroscopic investigation of maisine-based microemulsions as convenient carriers for co-delivery of anticancer and anti-inflammatory drugs

**DOI:** 10.1038/s41598-025-89540-w

**Published:** 2025-02-12

**Authors:** Mirela Nistor, Alina Nicolescu, Roxana-Maria Amarandi, Aurel Pui, Rares-Ionut Stiufiuc, Brindusa Dragoi

**Affiliations:** 1 Nanotechnology Laboratory, TRANSCEND Department, Regional Institute of Oncology, 2-4 General Henri Mathias Berthelot, Iasi, 700483 Romania; 2 Faculty of Chemistry, “Alexandru Ioan Cuza” University of Iasi, 11 Carol I Blvd., Iasi, 700506 Romania; 3 “Petru Poni” Institute of Macromolecular Chemistry, 41A Grigore Ghica Voda Alley, Iasi, 700487 Romania; 4 Department of Pharmaceutical Physics-Biophysics, Faculty of Pharmacy, “Iuliu Hatieganu” University of Medicine and Pharmacy, Pasteur 6, Cluj-Napoca, 400349 Romania

**Keywords:** Microemulsions, Liposomes, Fluorouracil, Ibuprofen, Spectroscopic investigations, Drug delivery, Drug delivery, Characterization and analytical techniques, Self-assembly

## Abstract

Lipid-based drug delivery systems are very promising in addressing critical medical needs associated with cancer because they are able to enhance the efficacy of the therapeutic agents loaded in. Yet, their transferability from bench to bedside is still a challenge as it hits many barriers. Among them, the absence of a clear design made on the deeper understanding of the intermolecular forces underlying the formation of the drug-carrier system and the controlled release of the drug is relevant. In this contribution, we rationally designed and prepared lipid-based formulations of an anticancer drug, fluorouracil (FU - hydrophilic) and an anti-inflammatory drug, ibuprofen (IBU - hydrophobic) to thoroughly characterize the specific intermolecular interactions between drugs and components of the carrier matrix. Microemulsions (ME) were selected as the main carriers for this study, but a comparison with liposomes was performed to observe if different organization of the lipophilic and hydrophilic compartments influences the loading capacity and controlled release of these two drugs. Using Maisine CC, a biocompatible oil, and Tween 20 as the surfactant, normal oil-in-water ME loaded with FU and IBU (1:1, 1:3, 1:6, wt:wt) were prepared by the water titration method. MEs were characterized by DLS, Zeta potential, and DOSY spectroscopies to assess their droplet size, surface charge, structure and type of emulsion. Intermolecular interactions between drugs and components of the ME’s matrix were investigated by FT-IR, RAMAN and ^1^H-NMR spectroscopies. The experimental results of DOSY revealed that all components of MEs are gathered in normal oil-in-water ME. Due to their different affinities for the main components of the ME, FU, and IBU were mainly distributed in the aqueous and oily phases, respectively, as supported by the droplet size measured by DLS. It was observed that co-loading the two drugs impacted the release behavior, assessed by the dialysis bag method, as compared with the mono-drug formulations. Based on the findings of this work, a release mechanism for FU and IBU was proposed, as well. Overall, the ME proved to be more suitable nanocarriers since the drugs, which were loaded in higher amounts as compared to liposomes, followed a controlled and sustained release of at least 96 h.

**Supplementary Information:**

The online version contains supplementary material available at 10.1038/s41598-025-89540-w.

## Introduction

The accidental discovery of liposomes in the ‘60s followed by the first clinical application of liposomal doxorubicin (Doxil) in 1995 has revolutionized the modern pharmaceutical formulations^1,2^. This attracted increasing interest in lipid-based drug delivery systems as carriers for anticancer therapeutic agents in both academic and industrial research. These carriers have many advantages, such as enhanced bioavailability, solubility, and controlled release of the therapeutic agents. Moreover, due to lipid components, they are especially interesting for drugs with low solubility in aqueous phase. Due to their well-delimited oil and aqueous compartments, such nanosystems can be simultaneously loaded with drugs of different solubility. On the other hand, anticancer drugs have poor chemical stability in the biological environment, undergo chemical degradation, exhibit systemic toxicity due to the lack of tumor selectivity, etc., requiring encapsulation in a carrier to solve these issues^3,4^.

Among the lipid-based carriers, microemulsions (MEs) attracted interest for drug formulation in the 1990s due to their easy preparation, even at a larger scale, from oil, water and an amphiphilic compound and a co-surfactant, if needed^5^. The size of the droplets is in the nano range providing a high surface for engulfing large amounts of drugs that can be then released in a controlled manner over time^6,7^. Moreover, their lipid composition allows for passively crossing biological barriers, enhanced absorption of drugs, increased bioavailability, and therapeutic efficacy^5,8^. They can be also decorated with specific targeting ligands, allowing for selective drug delivery to tumor sites^9^. All these advantages are solid premises for MEs as versatile carriers for tailored cancer therapy. So far, several antitumor agents have been loaded in biocompatible MEs, such as paclitaxel, methotrexate, curcumin, doxorubicin and sorafenib tosylate^10–15^. In terms of commercialization, only a few products are commercially available (Allermyl®, Solvium®, Sandimmune Neoral®, Norvir®, and Fortovase®)^5^, none for oncologic applications.

This current state clearly shows that MEs for medical applications are an area yet to be explored. However, many aspects have to be addressed in order to obtain a marketable product based on MEs. For instance, to understand their structural organization, the interactions between components, and generally their physico-chemical properties, a proper characterization is needed, but this is quite challenging^5^. Of particular interest for the design of proper drug formulations based on MEs is the study of the interactions between the drug and the ME’s components, which are defining for the cargo release behavior in the biological environment. In line with this, it was shown that the drug-lipid interactions can affect the structural stability of the vesicles, and modulate the therapeutic and toxic effects of drugs^16,17^. In addition, for ME, which requires surfactant for spontaneous formation of the droplets from triglycerides and water and their stabilization, understanding the drug-surfactant interaction is also important to modulate their physico-chemical properties and the performance of the final drug formulation.

In light of the above discussion, it is obvious that optimizing the physico-chemical properties of MEs for loading and delivery of anticancer drugs is based on the understanding of their physico-chemical properties, particularly the molecular interactions between the drug and the components of the ME and the diffusion properties of components, as reliable guarantors to advance the basic knowledge and ultimately the translation process towards the market/clinics. However, different ME can have different behaviors, which implies studies for each new ME-based nanodrug.

To get inside into the drug-matrix interactions, spectroscopic techniques are powerful tools to study them since they have access to the molecular vibrations of the bonds (FT-IR, RAMAN) and chemical shifts of protons due to electronic groups in intimate vecinity (^1^H NMR), thus providing detailed information on the molecular environment of the drug^18^. FT-IR analysis can detect modifications in the functional groups of both the drug and the components of the lipid-based carrier, providing key information about the formation of drug-carrier complexes or changes in the dynamic of the carrier upon encapsulation. For instance, Chaurawal et al. confirmed the formation of the sorafenib tosylate (a multikinase inhibitor that inhibits tumor growth and proliferation for the management of breast cancer)‑microemulsion system based on the typical fingerprints in the infrared spectrum^14^. Interactions of the hydrophobic tamoxifen, an anticancer drug, with lipid model membranes were studied using a lipid bilayer composed of 1-palmitoyl-2-oleoyl-sn-glycero-3-phosphatidylcholine (POPC) and 10 mol % of 1-palmitoyl-2-oleoyl-sn-glycero-3-phosphatidylglycerol (POPG)^19^. FT-IR results revealed that tamoxifen interacted with the lipid membrane through the hydrocarbon chain region exclusively, helpful information for the development of more effective drugs. As a complementary technique, Raman spectroscopy offers information about bonds that have low intensity in the infrared range, offering thus a more complete picture of the composition and the intermolecular chemical networks in carried molecule-ME systems^20^. NMR techniques, commonly employed to characterize micelles, have sparked interest in the characterization of more complicated systems, such as ME for drug delivery, as well^21,22^. Previous studies showed that worthwhile information may be obtained using NMR techniques applied to several of the most common nuclei found in the components of an ME, particularly ^1^H. NMR provides evidence on (i) the positions of molecules in the ME compartments and (ii) the state of drug molecules within the ME droplets, while the diffusion-ordered NMR spectroscopy (known as “DOSY”) is a great tool to discriminate between the molecular species based on their apparent translational diffusion coefficients. Therefore, this last method is critical to establish the type of ME loaded with drugs.

For this study, we proposed for the first time a drug formulation for cancer treatment by co-encapsulating an anticancer drug and an anti-inflammatory drug in a Maisine CC (glycerol monolinoleate)-based ME and investigated the specific intermolecular interactions between the drugs and the components of the ME’s matrix as well as their impact on the stability of the system and the controlled release of the drugs. In order to observe the impact of the organization of the lipophilic and hydrophilic compartments on the loading degree, distribution and release behavior of the two drugs, liposomes co-loaded with the same drugs were prepared by thin-film hydration, using egg phosphatidylcholine as lipid source. The rationale for selecting such drugs is to respond to the combination therapy, as well, as the cornerstone of cancer treatment^23^. Essentially, tumor growth is reduced by targeting two different mechanisms simultaneously, enhancing the therapeutic effect as compared to monotherapy. By loading an anticancer drug and an anti-inflammatory drug in the same ME, we target the inhibition of the DNA synthesis and the inflammatory process, since many types of cancer are associated with inflammation, as evidenced by tumor biopsies^24–26^. Hence, we have chosen 5-Fluorouracil (FU), which is the first-line therapeutic option for various types of cancer, such as colorectal, breast, pancreatic, and stomach cancers^27–29^ and ibuprofen (IBU)^30–33^, a non-steroidal anti-inflammatory drug. Several biocompatible MEs made of Maisine CC, Tween 20, and ethanol, as oil, surfactant, and co-surfactant, respectively were prepared by water titration method. The optimized ME was loaded with the FU and IBU at various mass ratios (1:1, 1:3, 1:6). Monocomponent drug formulations were also prepared and used as references. MEs were characterized in terms of droplet size, surface charge, structure, type of emulsion, and inter-/intramolecular interactions by DLS, Zeta potential, vibrational (FT-IR, RAMAN), ^1^H-NMR, and DOSY spectroscopies. The liposomes were characterized in terms of size and surface charge, loading degree and encapsulation efficiency. The controlled release of the drugs from both types of lipid-based carriers was assessed by the dialysis bag method followed by drug release quantification using UV-Vis and HPLC for FU and IBU, respectively. Free drug release was also performed to observe whether the delaying effect in the release was due to the dialysis bag pores. The experimental results of DOSY revealed that all components of MEs form a normal oil-in-water ME in which FU and IBU are mainly distributed in the aqueous and oily phases, respectively, depending on their solubility. Based on FT-IR and RAMAN results, specific interactions between the components of ME and drugs are established. The matrix of ME experienced slight structural changes during the controlled release tests, as ^1^H-NMR showed the presence of oil and surfactant residues in the release medium, favoring the release process. Yet, the quantification of the drugs was not affected by these residues, their controlled release occurring in line with their location in ME and solubility. The release of drugs from liposomes was not affected by the components of the carrier. Based on the experimental results, a mechanism of release from ME was also proposed.

## Experimental

### Chemicals and materials

Tween 20 (Sigma-Aldrich), ethanol (EtOH, Honeywell, 99,8%), fluorouracil (C_4_H_3_FN_2_O_2_, FU, Sigma, > 99%) as a model anticancer drug, ibuprofen (C_13_H_18_O_2_, IBU, TCI > 98%) as a model anti-inflammatory drug, phosphatebuffer (PB, a. K_2_HPO_4_ Sigma-Aldrich > 99%; b. KH_2_PO_4_ Lach:ner > 99%); distilled water (Thermo Scientific), acetonitrile (ROTH, HPLC grade), water (Honeywell, HPLC grade), o-phosphoric acid (Lach: ner 85%), chloroforme (CHCl_3_,VWR) were used without further purification. Chicken egg L-α-phosphatidylcholine (PC) was purchased from Avanti Polar Lipids Inc. Maisine® CC (Gattefossé) was a kind gift from Azelis Romania.

### Preparation of samples

#### Phase diagram for microemulsions and drug entrapment

Pseudo-ternary phase diagram was performed by water titration method and using oil (Maisine CC), surfactant (Tween 20): co-surfactant (EtOH) (S_mix_, 2:1, w/w %), and water. The mixtures of oil along with surfactant and co-surfactant were mixed in different weight ratios from 0.5:4.5 to 4.75:0.25. To define more precisely﻿ the phase boundaries in the phase diagrams, 10 different combinations of oil and S_mix_ were prepared: 0.5:4.5, 1:4, 1.5:3.5, 2:3, 2.5:2.5, 3:2, 3.5:1.5, 4:1, 4.5:0.5, 4.75:0.25. After mixing the oil and surfactant mixture, the ME was formed by dropwise water addition under magnetic stirring until a color change from a transparent to an opaque yellow was noticed. After establishing the composition of the stable formulation, the incorporation of the drugs, FU and IBU, in ME was performed by dissolving the drugs in the amount of water used for titration. Monocomponent (0.34 and 1% FU:1, 3, 6% IBU) and bicomponent drug (1% FU and 1, 3, 6% IBU) systems were prepared.

#### Liposomes

Liposomes were prepared through thin-film hydration (TFH)^34^, sonicated and subsequently extruded. In brief, lipids and IBU (6% with regards to final suspension volume) were dissolved in chloroform, which was slowly evaporated under reduced pressure. Lipid films were kept under vacuum, and traces of organic solvent were removed by flushing the system with N_2_. Dry lipid films were hydrated for 2 h with a 1% FU solution in 10 mM PB (pH 7.4) at 37 °C. The final lipid concentration in aqueous suspension was 20 mM. Initial drug-to-lipid ratios were 3.9:1 (w/w) for IBU and 0.65:1 (w/w) for FU. Sonication was carried out for 10 min at 26 kHz and 3% amplitude with an ultrasonic homogenizer (UP200Ht, 200 W, Hielscher Ultrasonics, Teltow, Germany, equipped with a S26d2 sonotrode). Extrusion was performed 11 times through 200 nm pore-size polycarbonate membranes using a Mini-Extruder (Avanti Polar Lipids Inc.) at room temperature. Unencapsulated drugs were removed through dialysis at 4 °C in PB. All prepared formulations were stored at 4 °C.

### Physico-chemical characterization of samples

*DLS and Zeta potential* After appropriate dilution with ultrapure water, AMERIGO Particle Size & Zeta Potential Analyzer and Vasco-Kin contactless remote optical unit from Cordouan (France) were used to measure the mean particle size and polydispersity index (PDI). For Zeta potential measurements a carbon electrode was used. All measurements were performed at 25 °C, with appropriate dilutions. Data were processed using the Smoluchowski equation.

*FT-IR spectroscopy* Infrared absorption spectra were recorded on a Jasco 660 Plus FT-IR spectrophotometer using KBr pellets as support for liquid samples. Spectra were recorded between 400 and 4000 cm^−1^, 16 acquisitions per spectrum, and a resolution of 4 cm^−1^.

*Raman spectroscopy* The Raman spectra were measured using the Renishaw™ inVia Reflex Raman (Renishaw plc, UK) confocal multilaser spectrometer at a resolution of ~ 1 cm^−1^. An internal silicon reference was used for calibration. The measurements were achieved using small amounts (~ 1 µL) of analytes; these analyte droplets were pipetted and left to dry directly on Raman transparent CaF_2_glass^35^. The 50× (N.A. = 0.75) objective lens was used to record the spectra. A 785 nm diode laser was used for excitation. The laser power (measurements relative to the sample surface) was ~ 65 mW while the acquisition time was set to 10 s. The spectrograph was equipped with a 1200 lines/mm grating and a charge-coupled device camera (CCD). Baseline correction was applied to all spectra to eliminate the fluorescence background. Each spectrum represents an average of a minimum of 30 spectral acquisitions, collected on different randomly chosen regions. The baseline correction was performed using the Wire 4.2 software provided by Renishaw.

^*1*^*H-NMR and DOSY spectroscopy* NMR experiments were performed on a Bruker Avance NEO 400 MHz spectrometer equipped with a 5 mm four nuclei (QNP-H, C, F, Si) direct detection, z-gradient probe. The DOSY experiments were recorded with ledbpgp2s standard TopSpin pulse sequence, using a diffusion time of 0.8 s and gradient pulse length of 2 ms. D_2_O was used as solvent for the NMR analysis.

*Phospholipid content* was quantified through a modified Stewart assay^36,37^. Briefly, 2 mL of aqueous 0.1 M FeCl_3_ and 0.4 M NH_4_SCN were added to a mixture of 10–20 µL liposomal suspension and 3 mL chloroform. The mix was vortexed for 1 min and centrifuged for 5 min at 2500 rpm, after which the lower organic phase was carefully transferred into a glass tube. Absorbance was read from the organic phase in quartz cuvettes at 469 nm. Determinations were made in triplicate. The calibration curve was composed of pure Egg PC, being linear in the 0–310 mol lipid range (R^2^ = 0.998).

*Encapsulation efficiency* (EE%) *and drug loading* (LD%) were determined after liposomes bursting with absolute ethanol and sonication for 10 min, following the removal of unencapsulated drugs. Drug concentration was determined spectrophotometrically at 266 and 221 nm for FU and IBU, respectively, using calibration curves specific for each drug. The encapsulation efficiency is expressed as:

$$EE\%=\frac{Encapsulated\;drug}{Total\;drug}\times100$$

where the *Encapsulated drug* represents the quantity of drug after liposome bursting, and the *Total drug* is the initial quantity of drug included in the formulation. *Drug loading* or *loading degree* is expressed as the weight of the encapsulated drug divided by the weight of liposomal formulation, after lipid concentration determination.

### In vitro drug release

Controlled release experiments were carried out by dialysis bag method using phosphate buffer solution (PB, pH 7.4) as release medium, at 37 °C and under gentle magnetic stirring (100 rpm, Heidolph MR Plug magnetic stirrer). The dialysis membrane (MW cut-off 14,000 Da, Sigma Aldrich) was loaded with 2 mL of sample containing the drug(s) of interest and submerged into 200 mL of release medium. At fixed time intervals, 2 mL of sample were withdrawn from the receptor medium to quantify drug release. They were immediately replaced with 2 mL of fresh buffer solution to maintain a constant release volume. IBU was quantified using a Dionex UltiMate 3000 UHPLC chromatographic instrument from Thermo Scientific, Bremen, Germany. The chromatographic conditions were as follows: Vydac analytical C18 column (4.6 × 250 mm); column temperature was room temperature; mobile phase consisted of acetonitrile and water (pH 2.7 with o-phosphoric acid) (60:40); flow rate was set at 0.3 µl/min, detection wavelength was at 221 nm, and the injected sample volume was 10 µL. To quantify the concentration of FU, UV-Vis absorption measurements were performed using a Shimadzu Ultimate-1900i UV-Vis spectrophotometer. The wavelength was set at 266 nm and the samples were transferred in a quartz cell with 1 cm path lengths. For IBU, the wavelength was set at 221 nm. Tests were performed in duplicate.

## Results and discussions

### Phase behavior and preparation of microemulsions

**Fig. 1:**
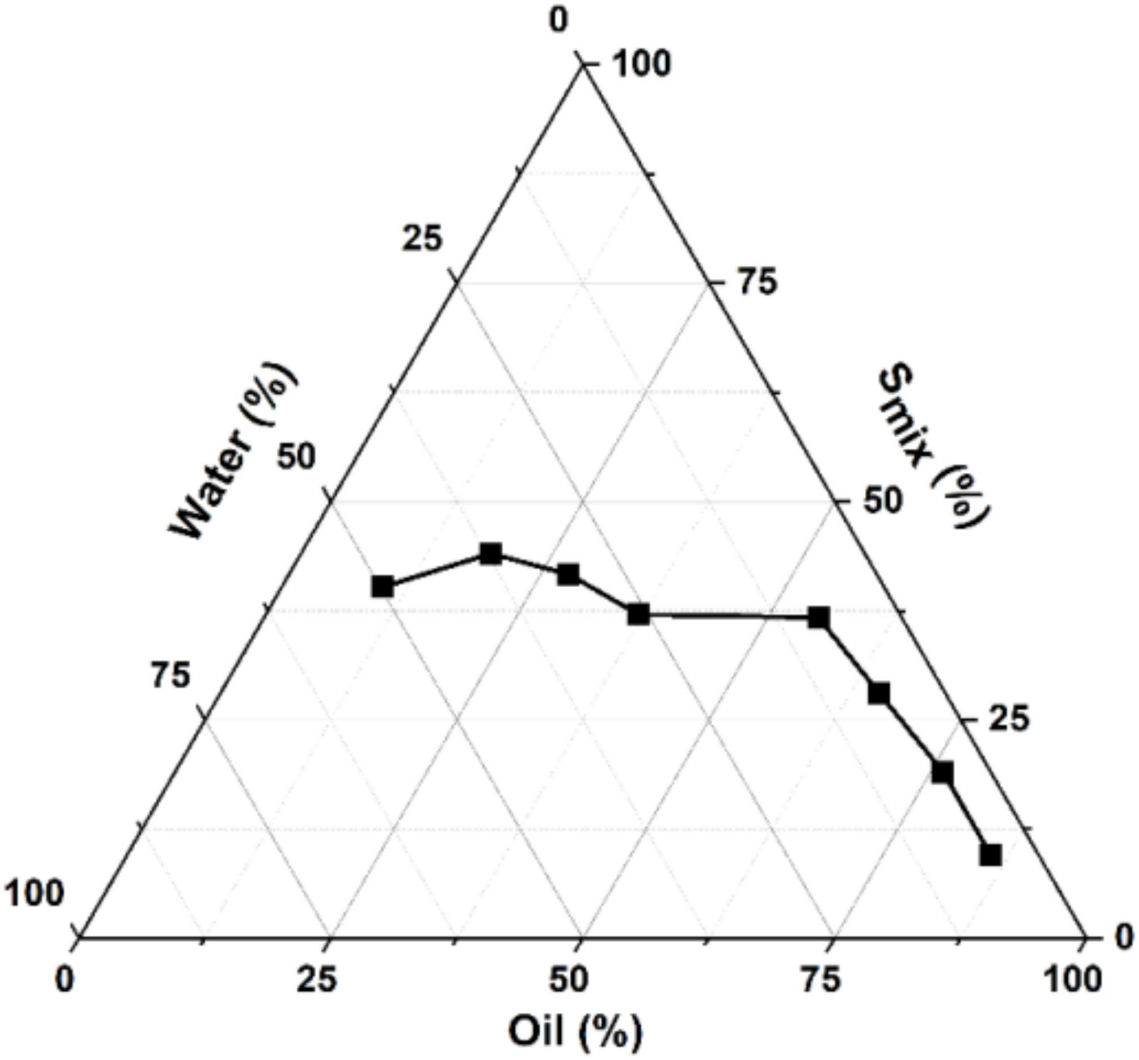
The pseudo-ternary phase diagram for the mixture Maisine CC–S_mix_–water.

Pseudo-ternary phase diagram (Fig. 1) was constructed according to Table 1, using Maisine CC as the oil phase, Tween 20 as the surfactant, and ethanol as the co-surfactant. Tween 20 is a non-ionic surfactant with a hydrophilic–lipophilic balance of 16.7, and due to this high value, it generally tends to form oil-in-water (O/W) MEs. Ethanol is a short-chain alcohol that decreases the interfacial tension in microemulsions and increases the interface’s suppleness. Due to their properties, these components are valuable for formulating microemulsions for drug delivery applications^38–40^. To obtain suitable concentration ranges of MEs components, a pseudo-ternary phase diagram was constructed in the absence of any of the drugs.

The phase study showed that the ME region narrows when the amount of S_mix_ decreases. The minimum quantity of oil that can be incorporated into microemulsion systems is 0.5 g. As the amount of S_mix_decreases, the volume of water needed to form the microemulsion increases. This may be due to the fact that in O/W MEs, surfactant molecules arrange themselves at the oil-water interface, with the hydrophilic head facing the aqueous phase and the hydrophobic tails facing the oil one. This arrangement of the surfactant molecule creates a molecular monolayer encapsulating oil droplets in the aqueous phase. It has been shown that by lowering the amount of surfactant, the monolayers can become spaced out, thus allowing more water incorporation into the system^41,42^.

**Table 1:** MEs compositions for phase diagram.

Sample	Composition		
	Oil (g)	S_mix_ (g)	H_2_O (g)
F1	4.75	0.25	-
F2	4.5	0.5	-
F3	4	1	4.95
F4	3.5	1.5	2.95
F5	3	2	2.2
F6	2.5	2.5	1.75
F7	2	3	0.45
F8	1.5	3.5	0.35
F9	1	4	0.25
F10	0.5	4.5	0.25

Formulations F1 and F2 (Table 1) did not form microemulsions because the surfactant concentration was deficient, and the number of surfactant molecules needed to reduce the interfacial tension for droplet formation was insufficient^43^. Of the ten formulations listed in Table 1, F9 was selected to encapsulate the drugs. The selection was made based on the ME’s stability over time (Fig. ES1). This formulation was used to prepare monocomponent and bicomponent drug-loaded MEs using FU and IBU as anticancer and anti-inflammatory, respectively, drugs. This combination was supported by the fact that tumors are many times accompanied by an inflammatory process and the addition of an NSAID to the therapeutic scheme was proved to potentiate the therapeutic effect of the anticancer agent^44^. Table 2 lists the final composition of the nine drug(s)-loaded MEs and the free drug MEs used in this study.

**Table 2:** Final composition of microemulsions.

Sample	Composition					Code in the paper
	Oil (g)	S_mix_ (g)	H_2_O (g)	FU (mg)	IBU (mg)	
ME_01	2.5	10	2.5	-	-	Pure ME
ME_02	2.5	10	2.5	51	-	0.34%FU_ME
ME_03	2.5	10	2.5	150	-	1%FU_ME
ME_04	2.5	10	2.5	-	150	1%IBU_ME
ME_05	2.5	10	2.5	-	450	3%IBU_ME
ME_06	2.5	10	2.5	-	900	6%IBU_ME
ME_07	2.5	10	2.5	150	150	1%FU_1%IBU_ME
ME_08	2.5	10	2.5	150	450	1%FU_3%IBU_ME
ME_09	2.5	10	2.5	150	900	1%FU_6%IBU_ME

### Encapsulation efficiency and drug loading

For MEs, the entire amount of drugs was incorporated due to the preparation method, which implied the addition of drug solutions to ME, without further washings. Therefore, the entire amount of drug(s) was incorporated into the MEs. On the other hand, liposomes exhibited a low encapsulation efficiency, with values of 3.18 ± 0.06% for FU and 3.05 ± 0.11% for IBU. Yet, the liposome bursting method can influence encapsulation efficiency results, and implicitly, the exact quantitative determination of encapsulated material, with methanol outperforming Triton-X in some cases^45^. At the same time, ethanol has induced rhythmic-pore formation in giant vesicles^46^ and outperformed methanol in disrupting vesicles^47^. The liposomes drug loading was calculated after damaging them with ethanol and limitations of this method are not excluded. Considering the greater initial drug-to-lipid ratio, drug loading was considerably higher for IBU, with a value of 17.61 ± 0.54%. This result is comparable to those reported in the literature for Egg PC-containing liposomes^48^. At the same time, drug loading for FU was 3.07 ± 0.06%, comparable to Mansoori et al.^49^. This suggests that while encapsulation efficiencies are low for both drugs, drug loading is higher for IBU in the expected range for liposomal vesicles.

### Physico-chemical properties

#### DLS

Zeta potential measures the surface charge of particles, whereas hydrogen bonds and van der Waals bonds determine the strength of attraction and repulsion between particles. It is accepted that the higher the zeta potential value, negative or positive, the more stable the systems will be. Usually, but not necessarily a rule, a low zeta potential value leads to an unstable system, which will eventually aggregate due to van der Waals interactions^50–52^. The values of zeta potential, average droplet size, and PDI measured for the MEs prepared herein alongside standard deviation are shown in Table 3. The zeta potential for the nine formulations has values between − 15.9 and − 28.98 mV, indicating very good stability of fresh MEs. Measurements performed after four months showed variations in size and Zeta potential, pointing out changes in the ME’s organization and stability, depending on the composition, particularly, the nature of the drug (Table 3). While for FU-loaded MEs an increase in size was noticed, the MEs loaded with IBU and FU-IBU exhibited better stability over time. In terms of zeta potential, the increase was more homogenous, inducing droplet aggregation. This observation can be explained if one considers the distribution of the two drugs in the emulsion. Indeed, the hydrophobic IBU should be located in the oil compartment of the ME while hydrophilic FU in the hydrophilic interlayer of the droplet (*vide infra*). In the case of monocomponent FU-based formulations, the zeta potential decreases from − 28.98 to −26.97 mV for an increase in the percentage of the FU from 0.34 to 1%, respectively. In contrast, for the monocomponent IBU-based formulations, the values increased with the drug’s percentage, with a difference of 6 mV between 1% and 6% IBU. In the co-encapsulated formulations, the zeta potential remains approximately the same, even if the ratio of the two drugs is changed.

DLS measurements showed that the particle size for the final compositions is between 115 and 257 nm. In the case of FU encapsulation, the particle size decreased compared to that of drug-free microemulsion. By contrast, for the IBU-based formulations, either monocomponent or bicomponent, the droplet size increases. Yet, the increase was more obvious for the monocomponent IBU-MEs. These results can be explained based on the solubility of the drug. The solubility of FU in Tween 20 is about 20 mg/mL, compared to its solubility in water, 12 mg/mL^53^. It can be hypothesized that FU is quite heterogeneously distributed in the ME, the most part being dissolved in the aqueous part of the ME while a very small part is distributed in the oil component of the ME. FU is thus most probably located at the interface between oil and water, leading to the reduction of the interfacial tension, which results in the decrease of the droplet size inside the system. On the other hand, considering the lipophilicity of IBU (302 mg/mL^54^) and low solubility in water (11 µg/mL^55^), it appears that it occupies the oil component causing droplet enlargement^56^. On the basis of this rationale, it can be affirmed that co-encapsulation of the two drugs in the MEs causes a quite clear distribution inside the MEs. Accordingly, the oil part contains the hydrophobic IBU while FU is located in both oil and water components, but preponderantly in the aqueous interface.

**Table 3:** The average droplet size (*n* = 3), zeta potential (*n* = 6), and polydispersity index (*n* = 3) for fresh samples and after 4 months.

Sample	Size, nm	StDev	PDI	StDev	Zeta potential, mV	StDev
Fresh samples						
Pure ME	139.3	1.62	0.21	0.03	−15.9	0.91
0.34%FU_ME	119.3	1.24	0.25	0.02	−28.9	0.06
1%FU_ME	115.4	0.45	0.27	0.01	−26.9	0.88
1%IBU_ME	143.0	3.19	0.26	0.03	−21.9	0.99
3%IBU_ME	257.6	5.07	0.34	0.02	−24.6	0.45
6%IBU_ME	194.6	0.20	0.29	0.02	−27.6	1.76
1%FU_1%IBU_ME	181.2	3.57	0.32	0.01	−22.8	0.50
1%FU_3%IBU_ME	178.1	3.91	0.28	0.02	−22.5	0.48
1%FU_6%IBU_ME	198.3	3.91	0.30	0.03	−21.5	0.72
After four months						
Pure ME	157.3	1.02	0.24	0.03	−21.6	0.92
0.34%FU_ME	176.9	3.21	0.32	0.001	−33.9	1.00
1%FU_ME	145.8	2.48	0.28	0.02	−33.2	0.84
1%IBU_ME	165.4	0.84	0.28	0.02	−30.1	1.66
3%IBU_ME	224.4	1.13	0.28	0.04	−28.6	0.73
6%IBU_ME	220.9	2.21	0.34	0.01	−27.1	1.13
1%FU_1%IBU_ME	196.6	2.11	0.29	0.01	−28.9	0.69
1%FU_3%IBU_ME	218.2	0.49	0.30	0.06	−24.3	0.34
1%FU_6%IBU_ME	199.3	2.21	0.32	0.01	−23.4	0.67

The polydispersity index indicates the heterogeneity/uniformity of droplet size distribution in MEs^57^. Usually, the values between 0.1 and 0.7 indicate a monodisperse formulation while values > 0.7 stand for a wider distribution of droplets, i.e., a polydisperse formulation. The values for the nine formulations included in this study range from 0.21 to 0.34, indicating a narrow and uniform droplet distribution (Table 3). For liposomes, the size after extrusion is ~ 173 (PDI 0.14) and 131 nm (PDI 0.14) for free and drugs-loaded liposomes while the Zeta potential is −33.57 and − 30.44 mV for free and drugs-loaded liposomes, respectively.

#### FT-IR

**Fig. 2:**
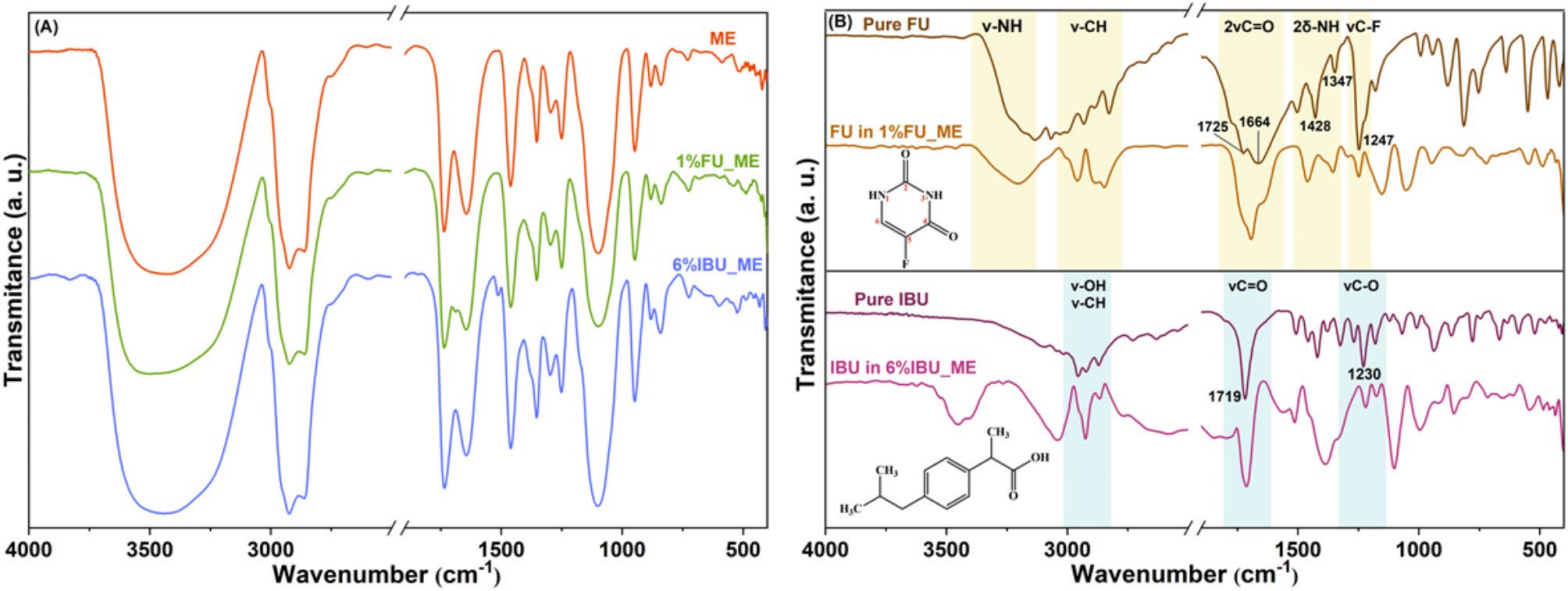
(**A**) FT-IR spectra of pure ME, 1%FU_ME and 6%IBU_ME. (**B**) FT-IR spectra: Pure FU and FU in ME (up), Pure IBU and IBU in ME (down).

The first vibrational analysis performed on the proposed formulations was FT-IR spectroscopy, aiming to identify the functional groups and their dynamics. FT-IR spectra of pure ME, 1%FU_ME, and 6%IBU_ME, as representative spectra of the series, are illustrated in Fig. 2.

As a first observation, no significant difference between the positions of the bands can be noticed irrespective of the composition (Fig. 2A). Yet, a change in the intensity of the bands can be observed in the spectra of 1%FU_ME and 6%IBU_ME samples, as compared to that of pure ME. This change could be related to the presence of the drug in the composition, but the bands of the two main components, that is, drug(s) and microemulsion, overlap. To shed more light on this aspect, the spectrum of the pure ME was subtracted from the spectra of the monocomponent drug-loaded MEs. The resulting spectra together with those of pure drugs are displayed in Fig. 2B. The subtracted spectra are complex, displaying either new or shifted vibrational bands. This may indicate the involvement of the functional groups in molecular interactions inside the ME. Moreover, the type of drug (i.e., FU or IBU) leads to spectra of various complexities, proof of different types of interactions depending on the nature of the drug. In Fig. 2B, only the bands specific to FU and IBU were highlighted in order to simplify the interpretation of the complex spectra. The spectra of pure FU and FU in ME, after ME spectrum subtraction, display bands in the 3500 –2600 cm^−1^ region, which are characteristic of the N-H and C-H stretching vibrations of the FU. The 1800 –1200 cm^−1^ region displays bands of the two C = O stretching vibrations groups (νC^2^ = O at 1725 cm^−1^ and νC^4^ = O at 1664 cm^−1^), the bending vibrations of the two N-H bonds (δN^3^-H at 1428 cm^−1^ and δN^1^-H at 1347 cm^−1^) and the stretching vibration of the C-F bond (νC-F at 1247 cm^−1^) specific to FU^58–60^. As noticed, band shifts are observed in the spectrum of FU in ME, but it is quite difficult to attribute this shift to a clear band as compared with the spectrum of pure FU. For example, the band at 1347 cm^−1^ is shifted by 8 cm^−1^ at higher wavenumbers, while a shoulder at 1390 cm^−1^ is noticed. This could indicate the involvement of this group in H bonding. The other N-H specific band is shifted by 32 cm^−1^ at higher wavenumbers and becomes broader, also indicating the formation of H bonds. At this stage, it is not evident with what component(s) of the samples these H bonds are formed. For the band of C-F, no shift is noticed in the spectrum of FU in ME, but only a decrease in intensity, most probably related to the relatively small amount of drug in the sample. It appears that C-F is not engaged in any interaction, neither intra- nor inter-molecular.

The spectra of pure IBU and IBU in ME after subtraction of ME display bands in the 3100 –2800 cm^−1^ region, which are characteristic of O-H and C-H stretching vibrations of the IBU (Fig. 2B)^61,62^. At 1719 cm^−1^, the well-defined carbonyl stretching of IBU can be observed, while in the spectrum of IBU in ME, this band is slightly shifted (7 cm^−1^) at lower wavenumbers. This shift could be the result of the involvement of the proton of the -COOH group in a H-bond. The stretching vibration corresponding to the C-O bond appears at 1230 cm^−1^ in pure IBU, whereas in the case of IBU in ME is shifted by 10 cm^−1^ (1220 cm^−1^).

#### Raman analysis

**Fig. 3:**
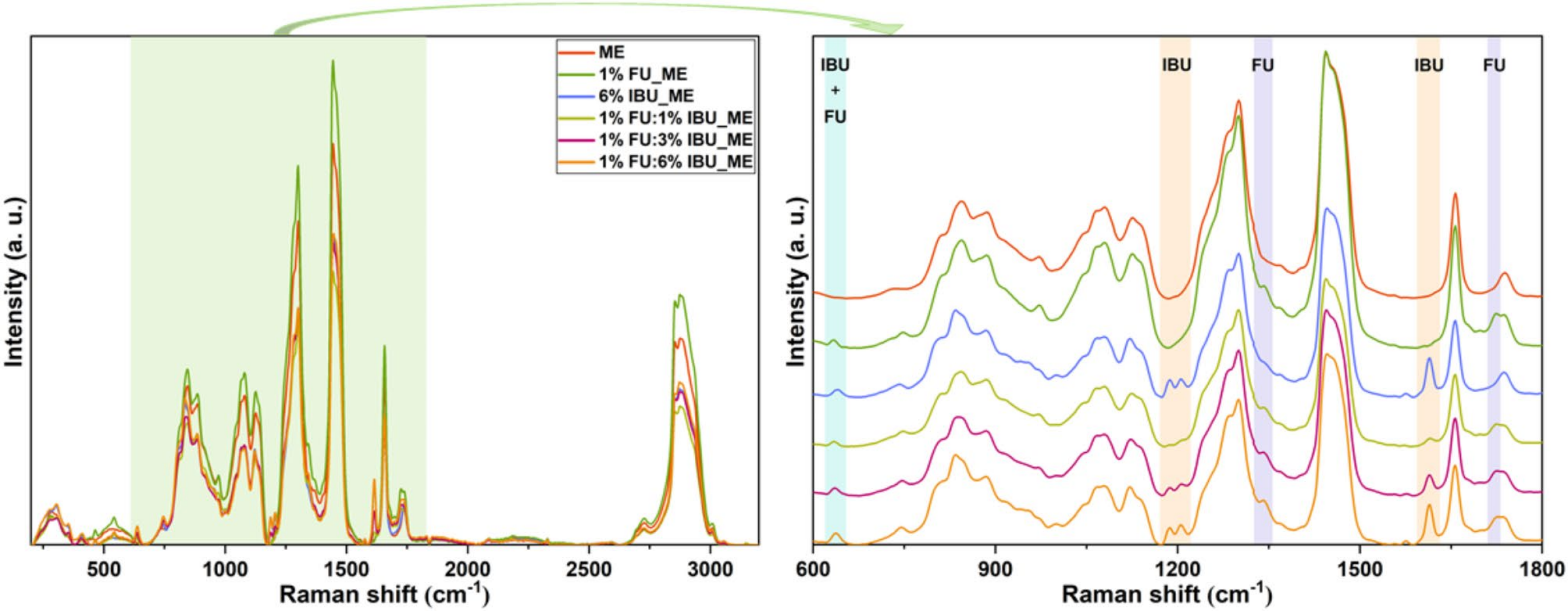
Raman spectra of pristine ME together with those loaded with FU, IBU and FU - IBU in different concentrations. The spectra were recorded using a 785 nm excitation wavelength.

It has been shown in the literature that the Raman spectrum of pure FU displays intense peaks at 790, 831, 1242, 1340, 1405, and 1673 cm^−1^^63^. These bands have been assigned to pyrimidine ring breathing (790 cm^−1^), trigonal ring + C-F vibrations (831 cm^−1^), trigonal ring + C-F stretching (1242 cm^−1^), ring + C-H wagging (1340 cm^−1^), ring + N-H wagging (1405 cm^−1^) and symmetric C = O stretching (1673 cm^−1^). Yet, the Raman spectrum of pure FU molecule is dominated by the doublet located around 800 cm^−1^ and the bands at 1346 and 1675 cm^−1^. The incorporation of FU in MEs prepared in this work can be demonstrated by the presence of two shoulders (~ 800 and ~ 1340 cm^−1^) and a very distinct peak at 1657 cm^−1^ in the recorded spectra (Fig. 3). These FU-specific bands are marked with light purple stripes in Fig. 3, right. In addition, peaks at 635 and 640 cm^−1^ for both drugs, FU and IBU, respectively, are displayed by the drug(s)-loaded MEs, which were attributed to C=C bonds, present in both molecules (aqua stripe in Fig. 3)^64^. In addition, a concentration-dependent increase in intensity is noticed for IBU.

 The 1187–1204 cm^−1^ doublet and the 1615 cm^−1^ peak represent IBU-specific bands^65^, which intensity increases in a concentration dependent manner, indicating its presence in the lipidic structure (light orange stripe in the figure above). In the bicomponent samples, the intensities of the IBU peaks generally decreases compared to the monocomponent samples, suggesting a competitive interaction between the two drugs.

#### NMR (^1^H/self-diffusion)

All microemulsions prepared in this work were analyzed by ^1^H-NMR and DOSY spectroscopy to structurally characterize the samples and to assess ME type (i.e., oil/water, bicontinuous, or water/oil), respectively^21,66^. The ^1^H-NMR profiles corresponding to individual components of MEs were first recorded. The ^1^H-NMR spectra for neat Maisine CC (oil) and Tween 20 (surfactant), together with the aqueous solutions of IBU and FU, are illustrated in Figs. ESI 2–ESI 6. For Maisine CC, it can be observed that most of the protons from the linoleate moiety resonate in the range of 0.5–2.6 ppm, with the signals of end chain CH_3_ groups at 0.61 ppm and middle chain CH_2_ groups at 0.99–1.02 ppm (Fig. ESI 1). The other four signals were assigned to the CH_2_ groups in different chemical environments, as follows: 1.31 ppm CH_2_ in beta position against carbonyl, 1.76 ppm CH_2_ in alpha position to the double bond, 2.02 ppm CH_2_ in alpha position against carbonyl and 2.47 ppm CH_2_ in between the double bonds. The protons of the double bonds resonate at 5.03 ppm. The glycerol moiety has resonance signals in the 3.25–4.80 ppm range. For the neat Tween 20 surfactant, the most intense signal is observed at 3.23 ppm, being assigned to CH_2_ groups of polyethylene glycol (PEG) moieties (Fig. ESI 2). The dodecanoate group has characteristic signals at 0.53 ppm (end chain CH_3_), 0.92 ppm (middle chain CH_2_ – 8 groups), 1.21 ppm (CH_2_ directly bonded to CH_3_), and 1.93 ppm (CH_2_ in alpha position against carbonyl). The signals for tetrahydrofuran residue are not visible in the proton spectrum, being overlapped by the intense signal assigned to PEG moieties. For pure IBU, the aromatic protons resonate as two doublets in the 7.07–7.15 ppm range (Fig. ESI 3). The signals at aliphatic protons give rise to five distinct signals in the range of 0.5–3.5 ppm, as follows: 0.75 and 1.26 ppm two doublets in the three CH_3_ groups, 1.71 (heptet) and 3.49 ppm (quartet) are attributed to the two CH groups, and 2.35 ppm corresponds to the doublet of the CH_2_group. FU gives only one signal in ^1^H-NMR, that is, a doublet at 7.55 ppm, which is attributed to the proton-fluorine couplings (Fig. ESI 4). Figure ESI 5 displays the ^1^H-NMR spectrum of pure ME. Besides the signals previously assigned to oil and surfactant, two new signals were visible at 1.28 ppm (triplet, CH_3_) and 3.72 ppm (CH_2_, quartet) assigned to ethanol. As oil and surfactant have several similar chemical fragments (e.g., OCH_2_ or long-chain CH_2_ groups), most of their proton signals are overlapped. Hence, only the signal at 5.43 ppm, assigned to the double bond protons, can be used as a “marker” for the oil phase. The water signal, which is of interest for diffusion, was assigned at 4.69 ppm.

**Fig. 4:**
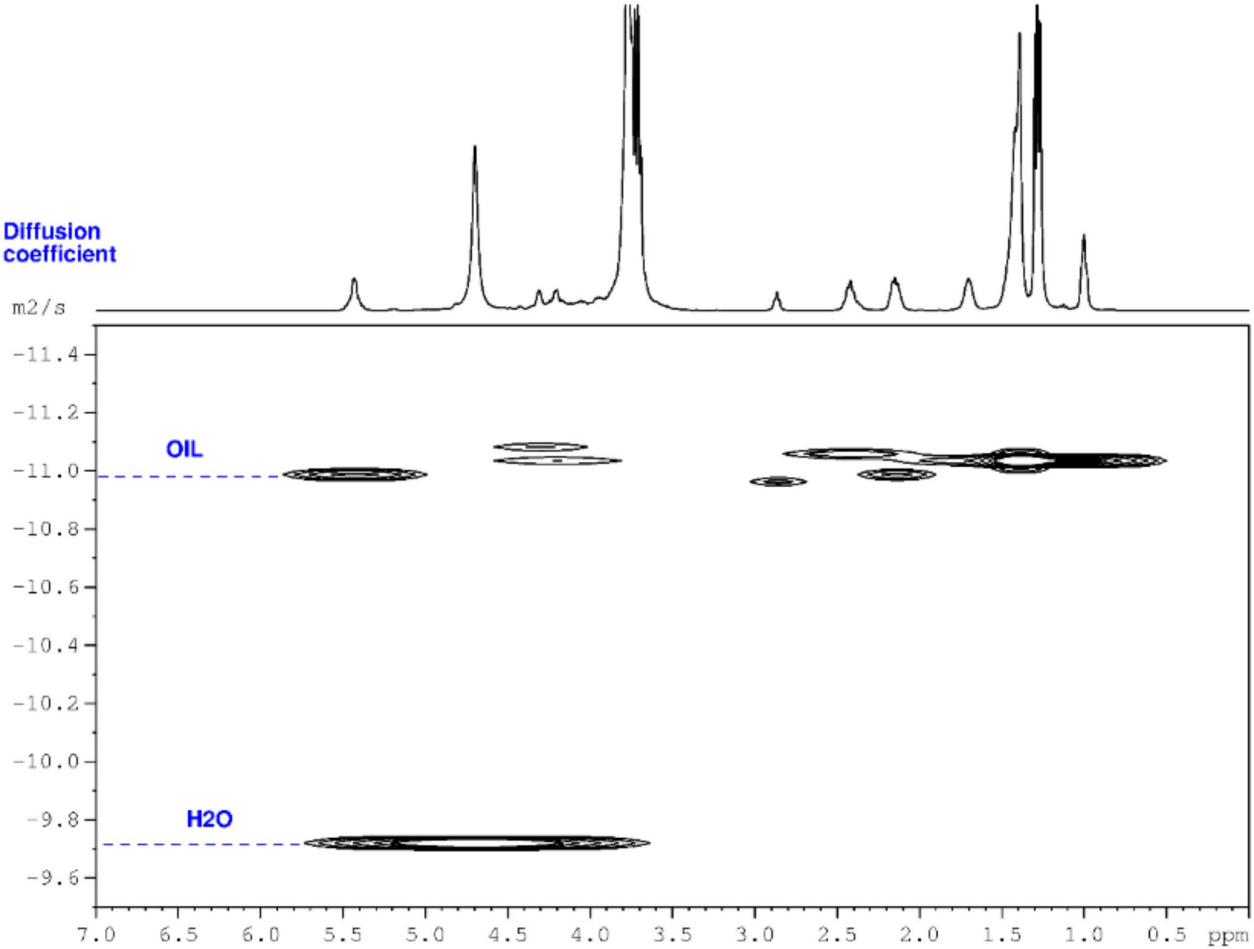
The DOSY spectrum of microemulsion showing a fast-diffusing water and a slow-diffusing oil and surfactant, recorded at 400 MHz.

The formulations used in this work have a ratio between oil and water of 1/1, which is challenging for establishing the type of ME., that is, O/W, W/O, or bicontinuous (Table 2). Therefore, DOSY spectra were recorded and the self-diffusion coefficients for water and oil in each sample were calculated. According to the literature, small droplets are formed if there is a difference of at least one order of magnitude between the self-diffusion coefficients of water and oil. By contrast, if the self-diffusion coefficients of the components are similar, a bicontinuous structure exists. Even more, the O/W or W/O microemulsion type can be determined by analyzing the difference between the self-diffusion coefficients of oil and water. Thus, if the self-diffusion coefficient of oil is much smaller than that of water, the microemulsion is O/W, the W/O microemulsion being the reverse case^21,66,67^. From the DOSY spectrum (Fig. 4), the oil (1 × 10^−11^ m^2^/s) and water (1.8 × 10^−10^ m^2^/s) diffusion coefficients were calculated.

**Fig. 5:**
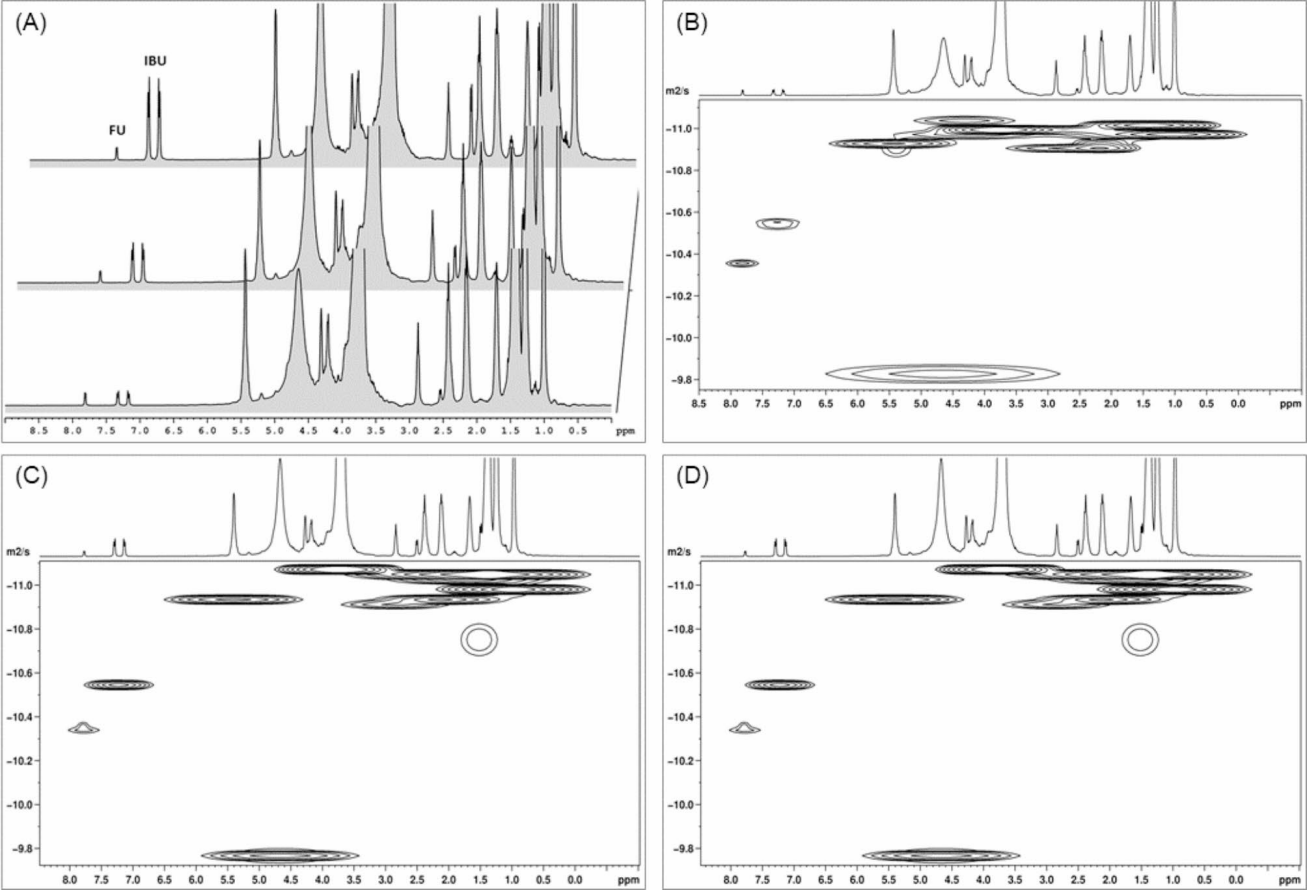
(**A**) ^1^H-NMR spectra of 1%FU:1%IBU_ME, 1%FU:3%IBU_ME, 1%FU:6%IBU_ME that confirms the presence of both FU (signal at ~ 7.5 ppm) and IBU (signal from 7.31 and 7.47 ppm) in the composition and the DOSY spectra for (**B**) 1%FU:1%IBU_ME, (**C**) 1%FU:3%IBU_ME, (**D**) 1%FU:6%IBU_ME recorded at 400 MHz.

Based on DOSY spectra and calculated self-diffusion coefficients, it was concluded that the type of microemulsions is O/W, irrespective of the composition. Representative proton and DOSY spectra recorded for the monocomponent drug-loaded MEs are depicted in Figs. ESI 7–ESI 11. For the bicomponent samples, the ^1^H-NMR and DOSY spectra are displayed in Fig. 5. The ^1^H-NMR spectra display signals of all the components of the emulsions. Furthermore, for the two drugs, it is obvious that the signals are dependent on the concentration of the drugs loaded in MEs. The signal of FU is constant in all spectra, while those of IBU increase as the concentration increases from 1 to 6%. The DOSY spectra were exploited to calculate the diffusion coefficients for all prepared MEs, that is, free, monocomponent and bicomponent in relation to the drug(s). The obtained values are displayed in Table 4. Apparently, no changes can be noted in the obtained values, suggesting the formation of the same type of ME for all samples, that is, normal O/W emulsion.

**Table 4:** Diffusion coefficients of the selected formulations.

Sample	Composition	Diffusion coefficient (m^2^/s)			
		Water	Oil (signal from 5.3 ppm)	FU	IBU
ME_01	Pure ME	1.8 × 10^−10^	1.0 × 10^−11^	-	-
ME_02	0.34%FU_ME	2.0 × 10^−10^	1.0 × 10^−11^	5.0 × 10^−11^	-
ME_03	1%FU_ME	1.5 × 10^−10^	8.6 × 10^−12^	3.6 × 10^−11^	-
ME_04	1%IBU_ME	1.7 × 10^−10^	9.4 × 10^−12^	-	2.5 × 10^−11^
ME_05	3%IBU_ME	1.6 × 10^−10^	9.7 × 10^−12^	-	2.5 × 10^−11^
ME_06	6%IBU_ME	1.7 × 10^−10^	1.0 × 10^−11^	-	2.5 × 10^−11^
ME_07	1%FU_1%IBU_ME	1.5 × 10^−10^	1.1 × 10^−11^	4.2 × 10^−11^	2.7 × 10^−11^
ME_08	1%FU_3%IBU_ME	1.7 × 10^−10^	1.1 × 10^−11^	4.2 × 10^−11^	2.7 × 10^−11^
ME_09	1%FU_6%IBU_ME	1.7 × 10^−10^	1.3 × 10^−11^	4.7 × 10^−11^	2.8 × 10^−11^

**Fig. 6:**
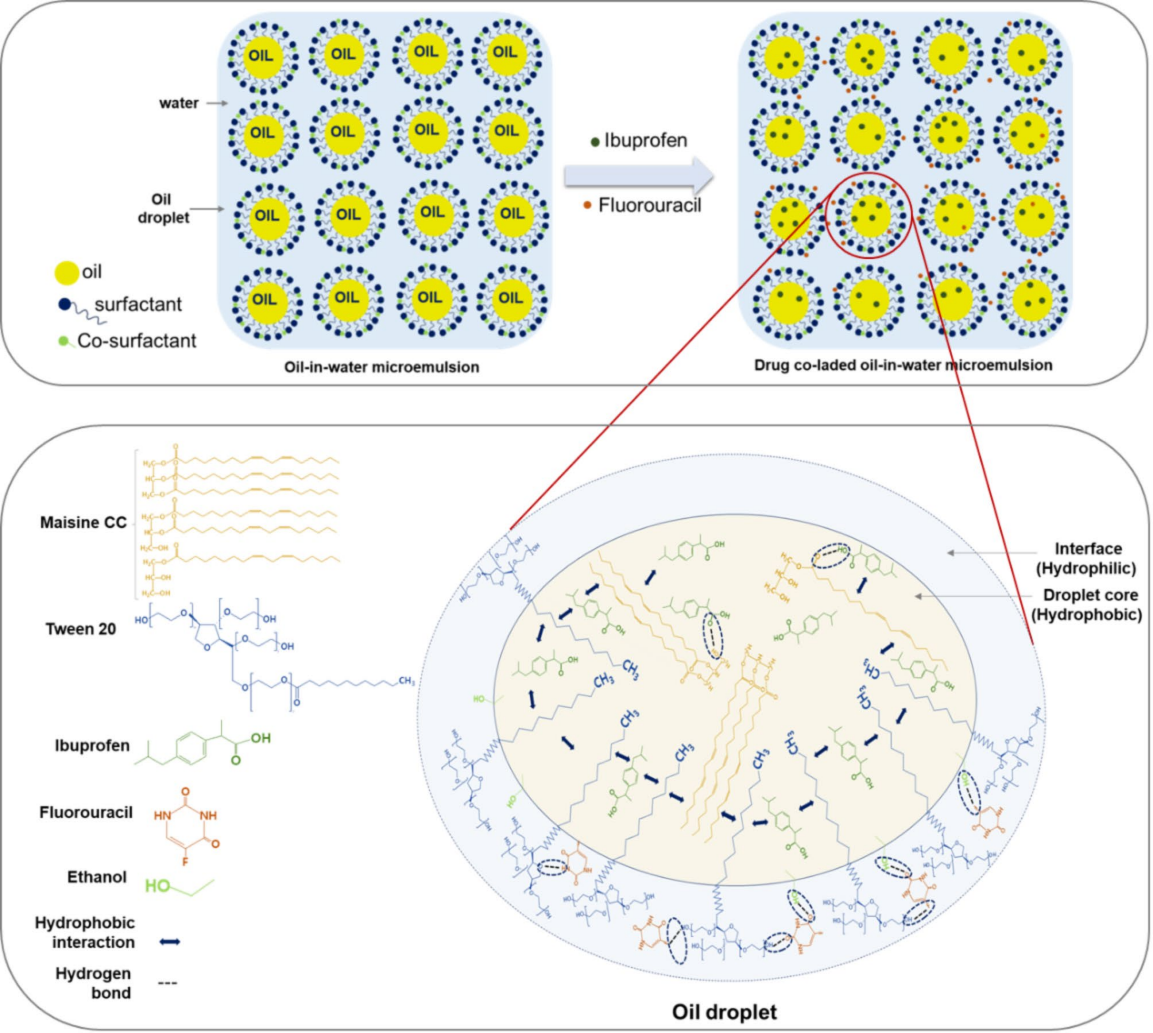
The proposed network of interactions of the components of the ME loaded with both drugs.

In line with the composition of the emulsions (Table 2) and the results of physico-chemical characterization, the structure of the MEs prepared in this work was proposed and depicted in Fig. 6. MEs are made of dense drops of oil separated from the aqueous environment by an interface made of the hydrophilic heads of the surfactant interconnected with the co-surfactant. When the drugs are incorporated, they are distributed in the components of the ME according to their solubility.

Based on the FT-IR and Raman analysis and considering that FU is a heterocyclic aromatic organic compound that contains strong hydrogen-bond donor (i.e., −NH) and acceptor (i.e., −CO) groups^68^, a network with possible drug - ME matrix interactions was proposed (Fig. 6). These groups allow intermolecular hydrogen bonds with the hydrophilic parts of the surfactant at the interface. Fluorine of FU is a hydrogen-bond acceptor^69^, which supports the involvement of an FU molecule in a maximum of five H bonds simultaneously. Yet, according to FT-IR results, it appears that FU does not establish any interaction with the ME components via F. On the other hand, the hydrophobic IBU is located in the core of the droplet, where is involved in neighboring hydrophobic interactions with the lipophilic tails of both surfactant and triglycerides.

#### Release test

**Fig. 7:**
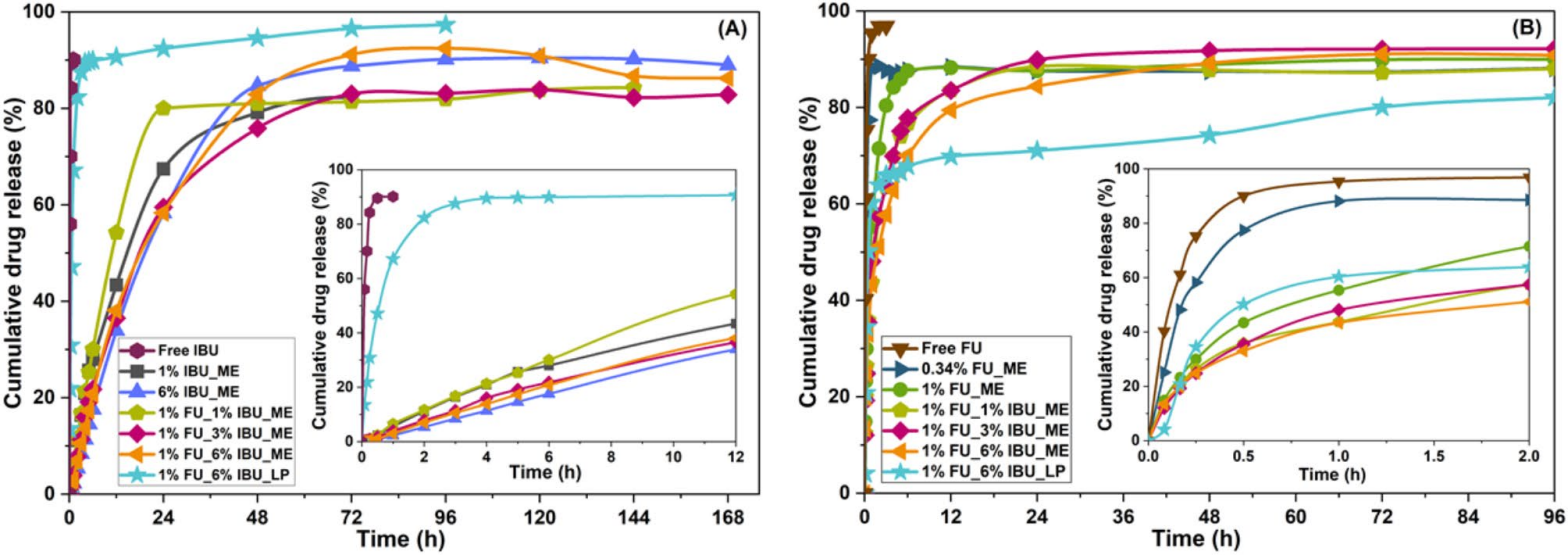
Drug release profile for IBU (**a**) and FU (**b**) from mono and bicomponent formulations.

The drug release behavior of the FU and IBU drugs entrapped in the MEs matrix was afterward assessed by the dialysis bag method at physiological pH, 37 °C, and gentile stirring. The obtained release curves are depicted in Fig. 7.

As a first observation, the drug release profiles of the two drugs indicate a much slower release of IBU than FU. Indeed, reaching a plateau of the released amount required 24 and 72 h for FU and IBU, respectively, afterward a sustained release until 48 and 168 h, respectively, was noticed. On the other hand, at least 60–85% of FU was released in the first 700 min of the test while for IBU, 25–58% was released in the same time interval. This behavior can be correlated with the distribution of the two drugs in the ME matrix depending on their solubility in the hydrophilic (for FU) and hydrophobic (for IBU) part of MEs and in the aqueous-based release medium. Therefore, it can be stated that drugs with various degrees of hydro/lipophilicity can be encapsulated in the ME formulations proposed in this work exhibiting different release behaviors. Interestingly, in the case of FU incorporated in the ME, different release behaviors were obtained depending on the amount of the drug. Hence, at 0.34% FU, a burst release was observed in the first 20 min of the test while ~ 85% of the drug was discarded in 60 min. Increasing the amount of the drug at 1% slowed down the release kinetics, with 25 and 50% of the drug being released in the first 20 and 60 min, respectively. The release of FU continued and reached a plateau at 85% in 360 min, instead of 60 min. Therefore, the release of FU from the sample with a higher amount was slower than that loaded with a lower amount of drug. Considering the heterogeneous distribution of FU in the aqueous phase of the ME - mostly at the surfactant/water interface of the drops - and the density of the drops, it can be assumed that for a low loading degree, no FU was placed in the core of the droplet, which facilitated a faster release. Once the amount of FU increased in ME, a part was entrapped in the internal compartment of the droplet. In this case, diffusion phenomena through the water phase controlled the release of FU from the internal part of the droplet to the surface and receptor environment. This hypothesis is reinforced by the similar release behavior of FU when discharged from the bicomponent samples. The profile is very similar to that of monocomponent 1%FU_ME. Moreover, a three-step FU release profile is obtained for the co-encapsulated samples, that is, < 60 min (step 1), 60–240 min (step 2), and > 240 min (step 3). In step 1, the release of ~ 40% of FU occurred followed by a delayed release of FU up to 60–70%, and finally, the released amount reached 70–80% in 720 min. Note that the kinetic profile of the release in step 2 is changed, maybe due to slight droplet deformation due to the simultaneous release of IBU from the oil droplet.

For IBU monocomponent formulations, the time-dependent release is much slower as compared to FU and much longer release periods are required, irrespective of drug concentration. It is obvious that in this case, a much-improved control of the release was achieved. Thus, ~ 20% of the total loaded IBU was released in a controlled manner in 360 min while almost 90% was discarded in 5760 min (96 h), followed by sustained release. IBU is a hydrophobic drug and thus, it is distributed in the oil core of the ME, surrounded by water. The release of IBU is thus a more complex process because it has to go out of the dispersed oil droplet followed by the passage through the continuous aqueous phase, to reach the receptor medium. In the bicomponent formulations, the release of IBU follows the same trend as in the absence of FU. This indicates an independent release behavior with no interaction with the anticancer drug (*vide supra*). However, this behavior is different from that of FU in the bicomponent samples. Therefore, the release behavior of these two drugs cannot be associated with an intermolecular interaction between them, but with diffusion phenomena.

**Fig. 8:**
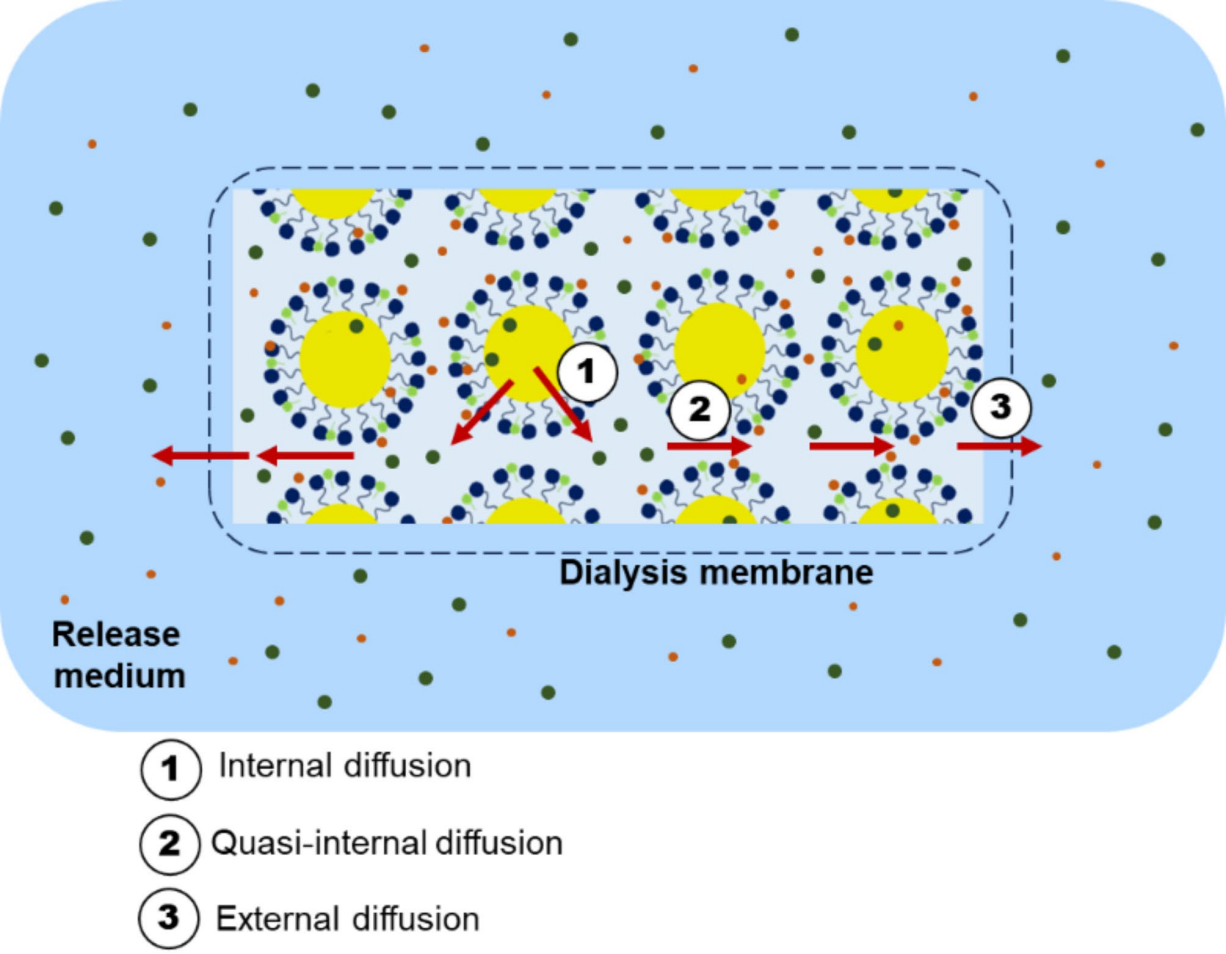
Release of FU and IBU from MEs.

Overall, the drug release from ME to the receptor medium is a 2- and 3-step process for FU and IBU, respectively (Fig. 8):

1. Diffusion from inside the oil droplet to the dispersing phase through the interfacial layer, which is made of hydrophilic heads of the Tween-20 and ethanol OH groups (*internal*).
2. Diffusion through the aqueous dispersing phase (*quasi-internal*).
3. Diffusion from the dispersing phase to the receptor medium through the dialysis membrane (*external*).

It should be underlined that FU diffusion is faster, particularly because most parts of the molecules are placed in the interfacial layer, whereas for IBU, diffusion is assumed to occur in three steps. The first one is the diffusion from the core to the interface, which should occur at a high rate due to the hydrophobic environment. Once the molecule reaches the interface zone, and then the aqueous dispersing phase, the diffusion is expected to be much slower due to the hydrophilic environment. The passage of IBU through this aqueous phase is the rate-controlling step of the general kinetic drug release. Indeed, the release rate of IBU is much slower while the release profile of FU indicates a faster release.

As mentioned in the Experimental section, the monitorization of FU release was made by UV-Vis absorption measurements performed at 266 nm while that of IBU by HPLC at 221 nm. Initially, the monitorization was performed by UV-Vis for both molecules, but quantification of IBU led to overestimated amounts of discharged IBU. In order to clarify this point, simulation of the controlled release from empty MEs was performed and the corresponding UV-vis spectra were recorded for samples taken off at the same time intervals as for the drug-loaded ME. Figure ESI 12 displays the UV-Vis absorption spectra of unloaded MEs samples while the UV-Vis spectra of FU and IBU are depicted in Figs. ESI 13 and ESI 14. As noticed, the absorbance of the MEs’ components overlaps that of IBU. Yet, this did not happen for FU, whose absorption occurs at 266 nm. It is obvious that the release of drugs is accompanied by the release of ME’s components, which partially collapse under the biological simulating experimental conditions, favoring the drug release. To confirm the presence of these components, ^1^H-NMR of samples taken off at various time intervals for the controlled release tests of FU-IBU-loaded MEs was performed and the results are displayed in Fig. ESI 15. Therfeore, to exclude the experimental errors from the calculations, the quantification of released IBU was performed by HPLC.

When the release of these two drugs was performed from liposomes, a burst release was noticed for IBU in the first hour of the test while the plateau was reached in almost three hours. This behavior could be explained by the lower amount of IBU retained in the phospholipid bilayer as compared with the ME. Yet, when the comparison is made with an ME in which a low amount of IBU was encapsulated (1%), the release behavior is similar to that of the samples with 6% IBU. Therefore, the faster release of IBU from liposomes cannot be explained by the amount of drug in the sample, but rather by a faster diffusion of IBU through the bilayer. Therefore, the encapsulation of IBU in a ME appears to be more suitable if a slow release is required. On the contrary, the release curve for FU has a S-shaped profile, showing a very small release in the first five minutes, followed by a release similar to that of FU in ME in the first two hours. In this period, ~ 60% of FU is discharged from liposomes, followed by a sustained release of FU up to ~ 70% in 48 h. After this point, a small jump in the FU release is noticed, followed by a new phase of sustained release up to ~ 80% in 96 h. It can be noticed that unlike IBU, which was completely discharged from liposomes, FU was not totaly discharged for the same release time. The results are particularly worthwhile because when FU was released from liposomal monodrug formulation, the behavior was different, the drug being expelled much faster^70^. On the other hand, the experiments of controlled release of free drugs indicated a burst release of the entire amount of drug in a very short time (Fig. 7), clearly pointing out that the release is not a phenomenon controlled by the pore of the dialysis membrane, but is truly a release controlled by the ability of the carrier to discharge the payload.

## Conclusions

To conclude, this work provides compelling evidence that first-time reported Maisine CC-based MEs co-encapsulating a hydrophilic (FU) and a hydrophobic (IBU) drug, hold promise as carriers for cancer combination therapy. The work was focused on the study of the physico-chemical properties of the fresh formulations, with a particular emphasis on the intermolecular interactions between the drugs and the carrier. Their long-term stability as a critical aspect in the translation process was investigated, as well. The experimental results of DOSY spectroscopy revealed the formation of a normal O/W ME while those of FT-IR, RAMAN, and ^1^H-NMR, showed the molecular interactions between the drugs and the ME components, suggesting that hydrogen bonding and hydrophobic interactions sustain the structural integrity of the formulations. The stability of the formulations was correlated with the zeta potential and droplet size of the MEs, with formulations containing both IBU and FU showing larger droplets. These formulations were stable for four months at 4 °C. In order to evaluate how the structural organization of a lipid-based carrier impacts the drug loading degree and the release behavior, a comparison with a liposome, with an opposite organization of the hydrophilic/hydrophobic compartments, was considered, as well. This comparative study showed that the drug loading degree was higher in ME than in liposomes. Then, it was interesting to see not particularly the distribution of the FU and IBU at the oil-water interface and oil phase, respectively, in the two types of lipid-based carriers, but their release behavior from the two types of nanosystems. Therefore, the release of IBU from ME was not influenced by the presence of FU in the formulation while the release of FU from ME in the presence of IBU was delayed. More interestingly, the release of two drugs from liposomes was significantly changed, with a faster release of IBU and a much slower and more sustained release of FU. The results are particularly significant for FU, which is usually faster discharged from monocomponent formulations. The experimental findings of this work clearly show the essential role played by the structural organization of a lipid-based carrier in controlling the release behavior of drugs. Also, co-loading two drugs with different solubility and affinity for the oil and water phases appears to be of equal importance in modulating the release behavior of a drug in the presence of the second one. Hence, the holistic analysis of the two systems, ME and liposomes, points out MEs as better carriers for FU and IBU, as representative molecules for drugs with different solubility profiles. This is crucial for designing lipid-based carriers as promising candidates for the translational process. Equally important, this work showed that Maisine CC-based MEs are a feasible platform for the co-delivery of hydrophobic and hydrophilic drugs, with high potential in cancer treatment. It can be stated that by providing insight into the physico-chemical properties, release mechanisms, and long-term stability of this proposed drug formulation, this work added scientific values to the current knowledge, being of paramount importance for the applicability of Maisine –CC- based ME in the medical field.

## Electronic supplementary material

Below is the link to the electronic supplementary material.

Supplementary Material 1

## Data Availability

Data and images used herein are found in the article and ESI. They are available from the ca. only in line with the confidentiality arrangement and based on strongly and realistic justified request.

## Abbreviations

EtOH: Ethanol
FU: Fluorouracil
IBU: Ibuprofen
PB: Phosphate buffer
PBS: Phosphate buffer saline
PC: L-α-phosphatidylcholine
