# Supplementary material for: Multi spectroscopic investigation of maisine-based microemulsions as convenient carriers for co-delivery of anticancer and anti-inflammatory drugs
Source: Sci Rep. 2025 Feb 12;15:5175. doi: 10.1038/s41598-025-89540-w (PMC11822113; doi:10.1038/s41598-025-89540-w)
Supplement: Supplementary file 1 — Supplementary Material 1 [file 41598_2025_89540_MOESM1_ESM.docx]

**Supplementary Information**

**Multi Spectroscopic Investigation of Maisine-based Microemulsions**

**as Convenient Carriers for co-Delivery of Anticancer and**

**Anti-inflammatory Drugs**

Mirela Nistor^1,2^, Alina Nicolescu^3^, Roxana-Maria Amarandi^1^, Aurel Pui^2^, Rares-Ionut Stiufiuc^1,4^, Brindusa Dragoi*^1,2^

1. Nanotechnology Laboratory, TRANSCEND Department, Regional Institute of Oncology, 2-4 General Henri Mathias Berthelot, 700483 Iasi, Romania
2. Faculty of Chemistry, „Alexandru Ioan Cuza” University of Iasi, 11 Carol I Blvd., 700506, Iasi, Romania
3. "Petru Poni" Institute of Macromolecular Chemistry, 41A Grigore Ghica Voda Alley, 700487 Iasi, Romania
4. Department of Pharmaceutical Physics-Biophysics, Faculty of Pharmacy, "Iuliu Hatieganu" University of Medicine and Pharmacy, Pasteur 6, 400349 Cluj-Napoca, Romania

* Corresponding [brindusa.dragoi@yahoo.com/ transcendbd@iroiasi.ro](mailto:brindusa.dragoi@yahoo.com/%20transcendbd@iroiasi.ro)


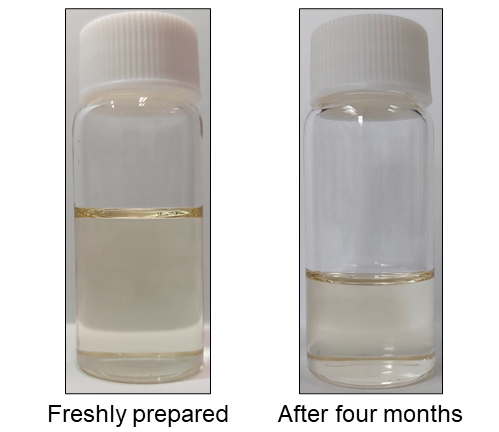


**Figure ESI 1.** Representative images of fresh ME and after four months of storage at 4 ˚C.


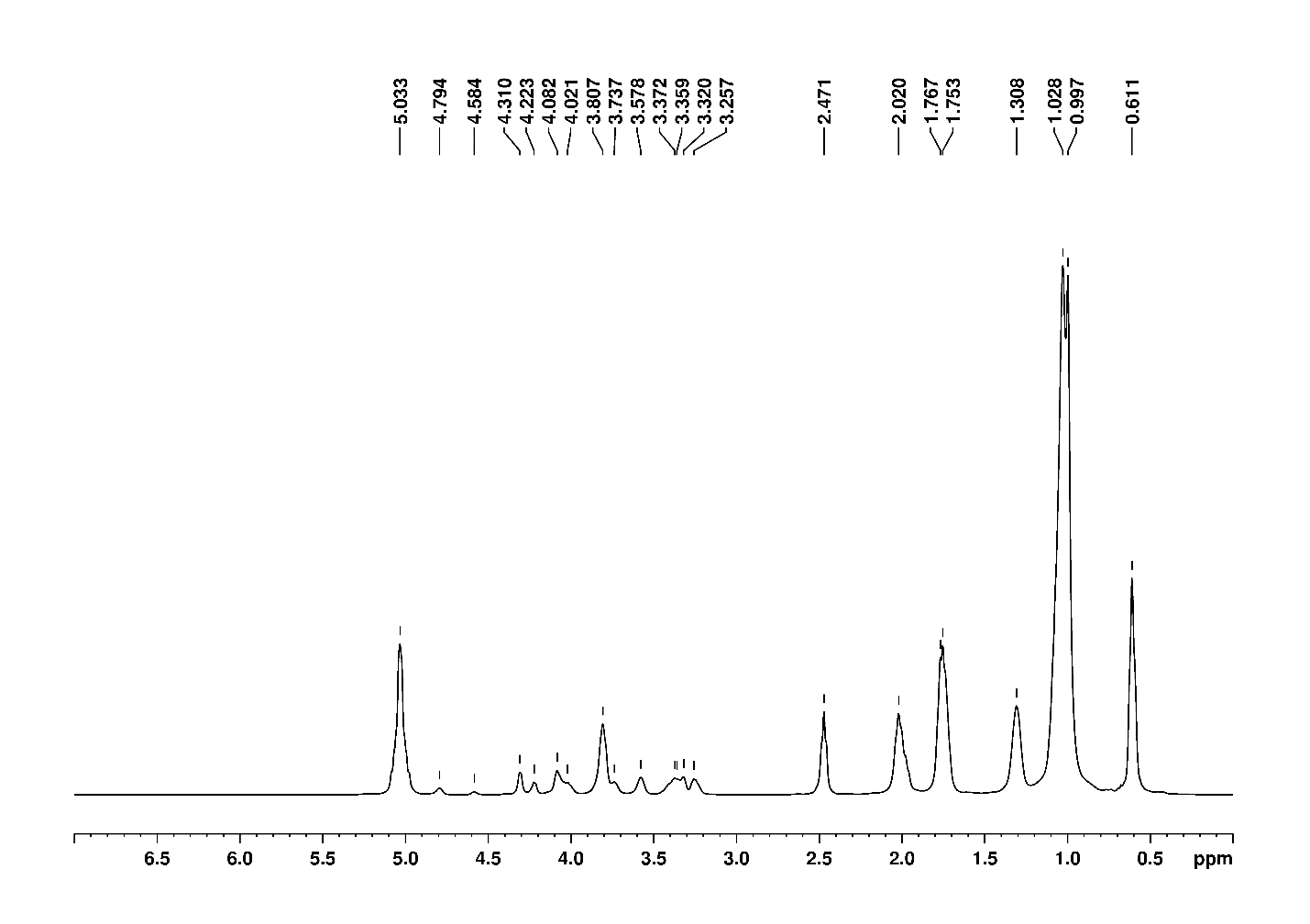


**Figure ESI 2.** The ^1^H-NMR spectrum of neat Maisine CC, recorded at 400 MHz.


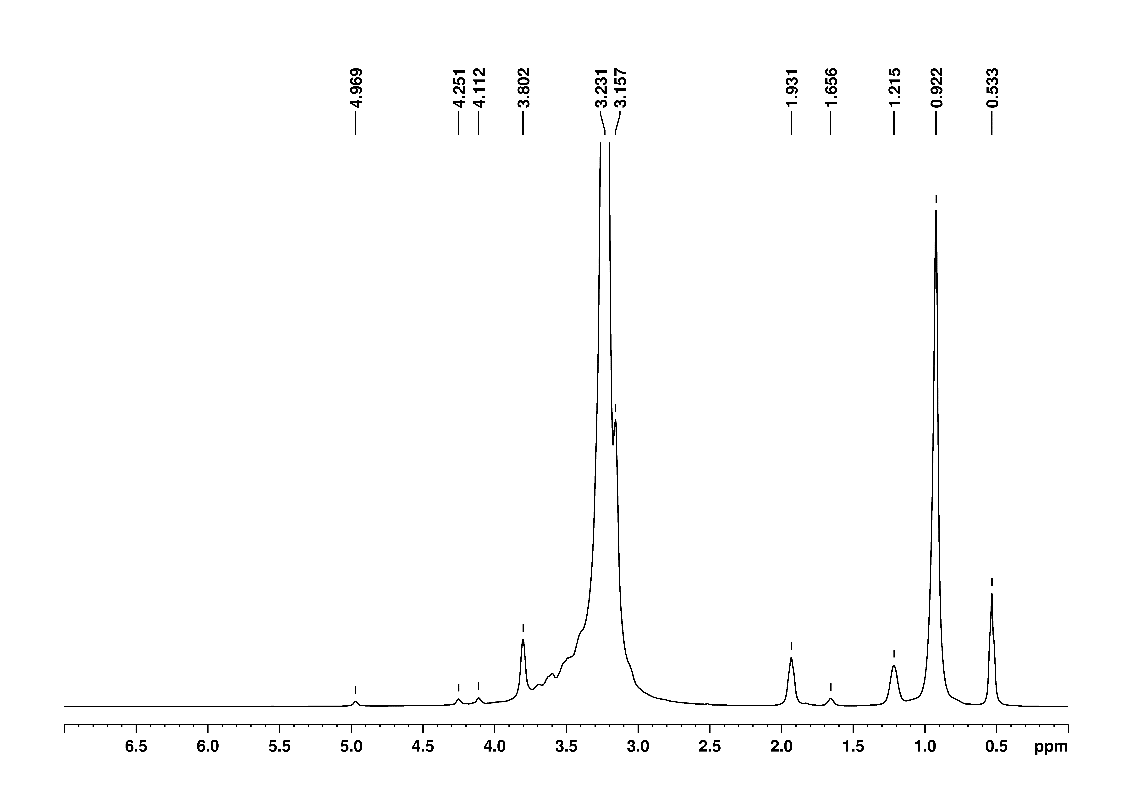


**Figure ESI 3.** The ^1^H-NMR spectrum of neat Tween 20, recorded at 400 MHz.


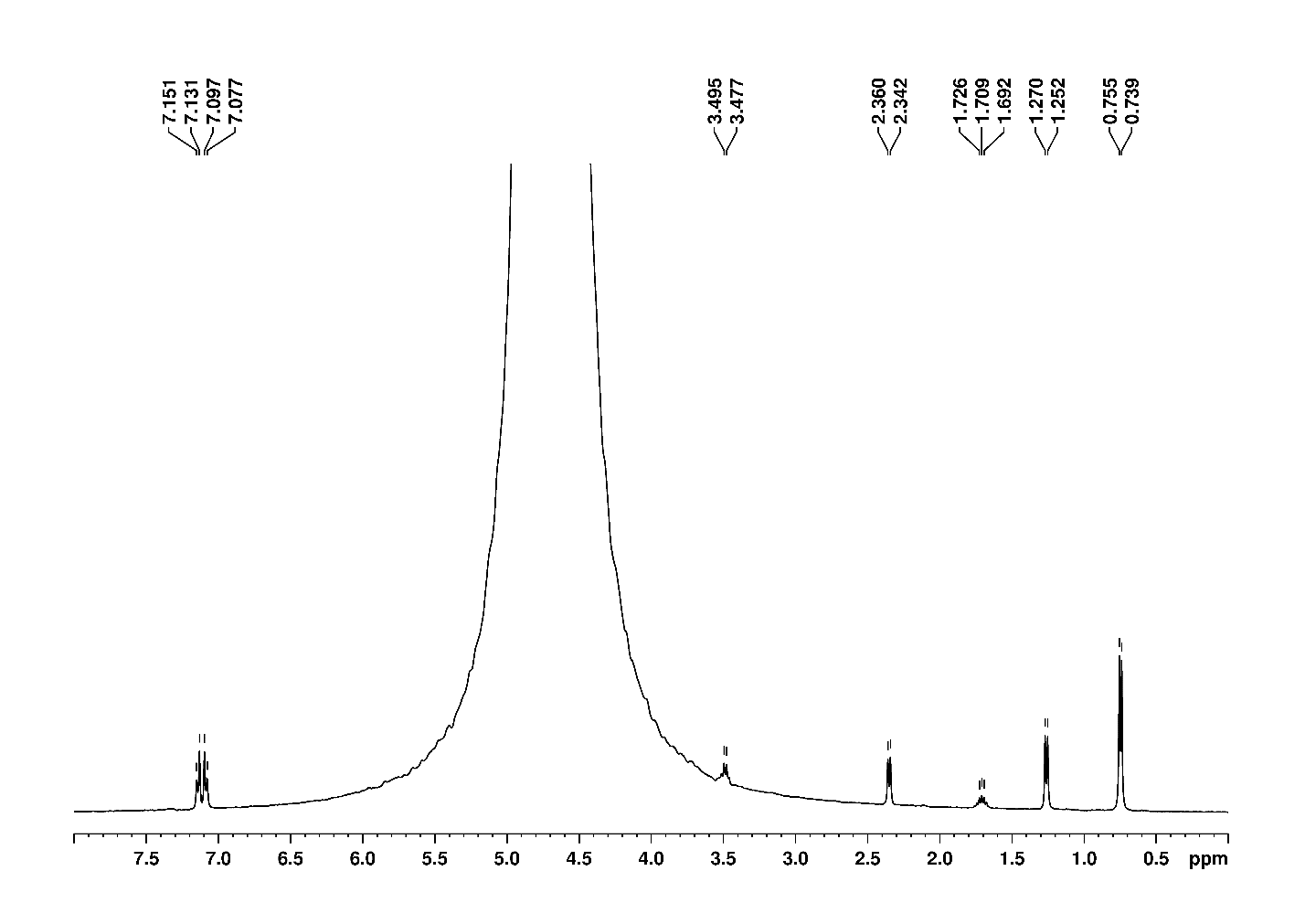


**Figure ESI 4**. The ^1^H-NMR spectrum of ibuprofen aqueous solution, recorded at 400 MHz.


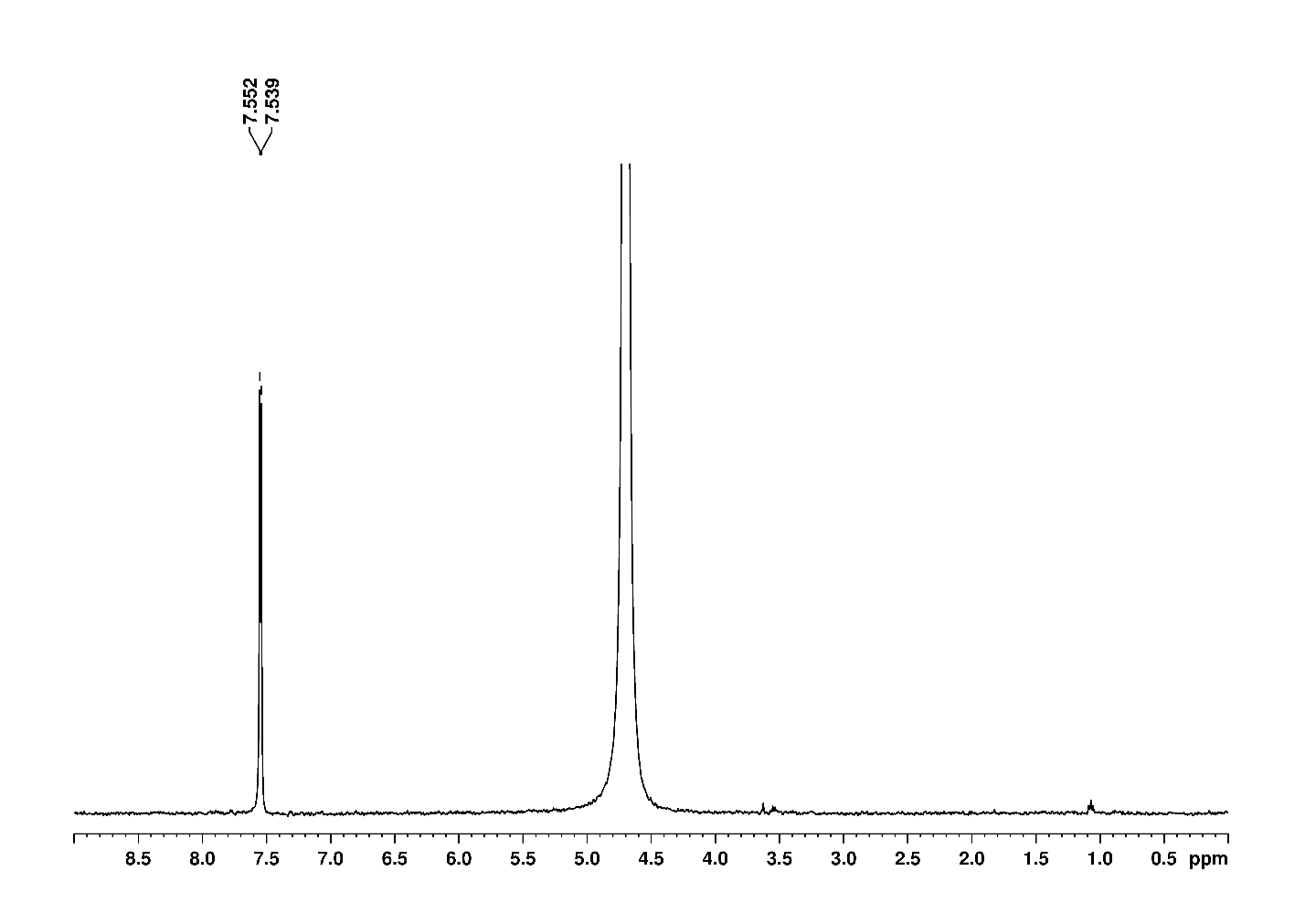


**Figure ESI 5**. The ^1^H-NMR spectrum of 5-fluorouracil aqueous solution, recorded at 400 MHz.


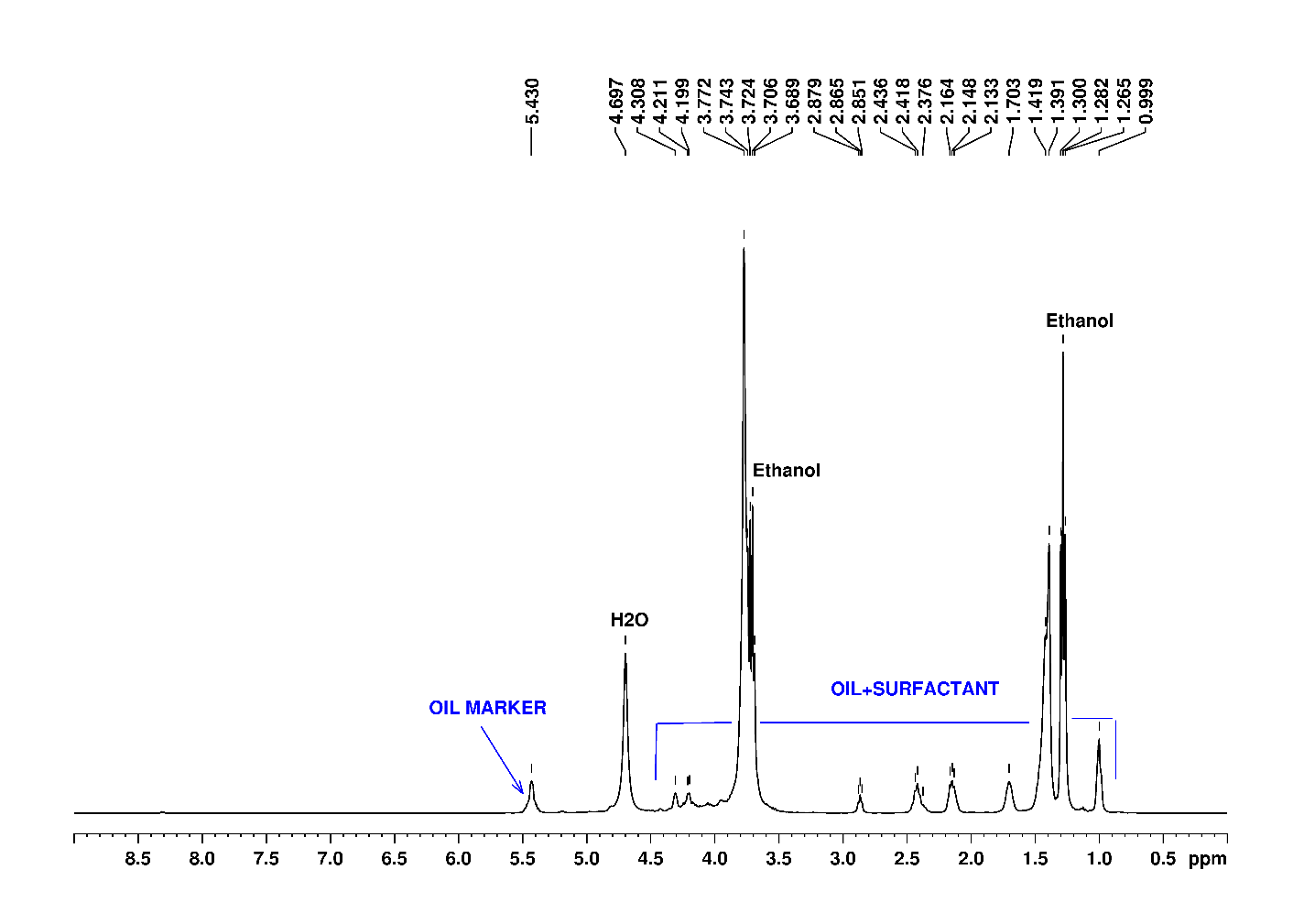


**Figure ESI 6**. The ^1^H-NMR spectrum of pure ME recorded at 400 MHz.

**A)**
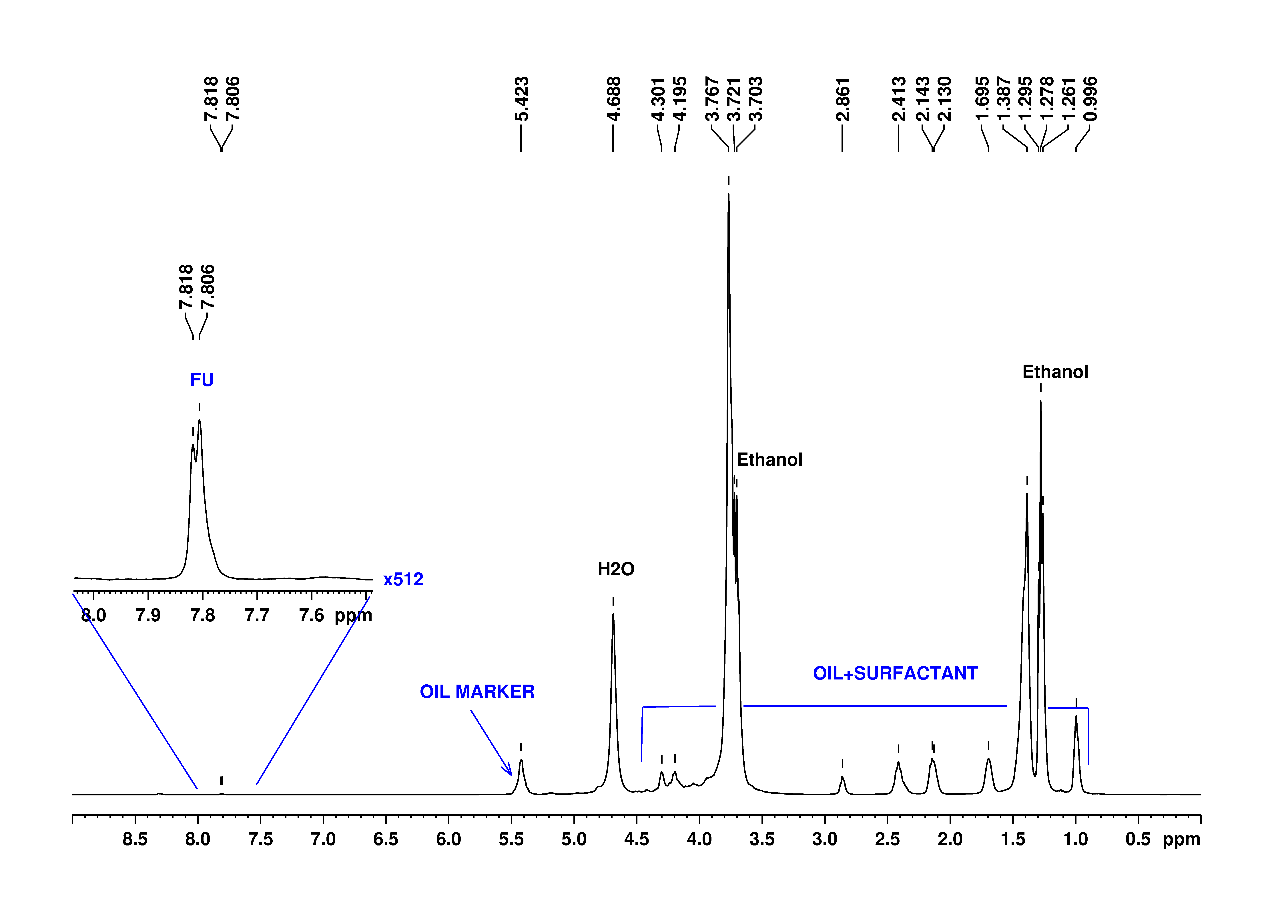


**B)**
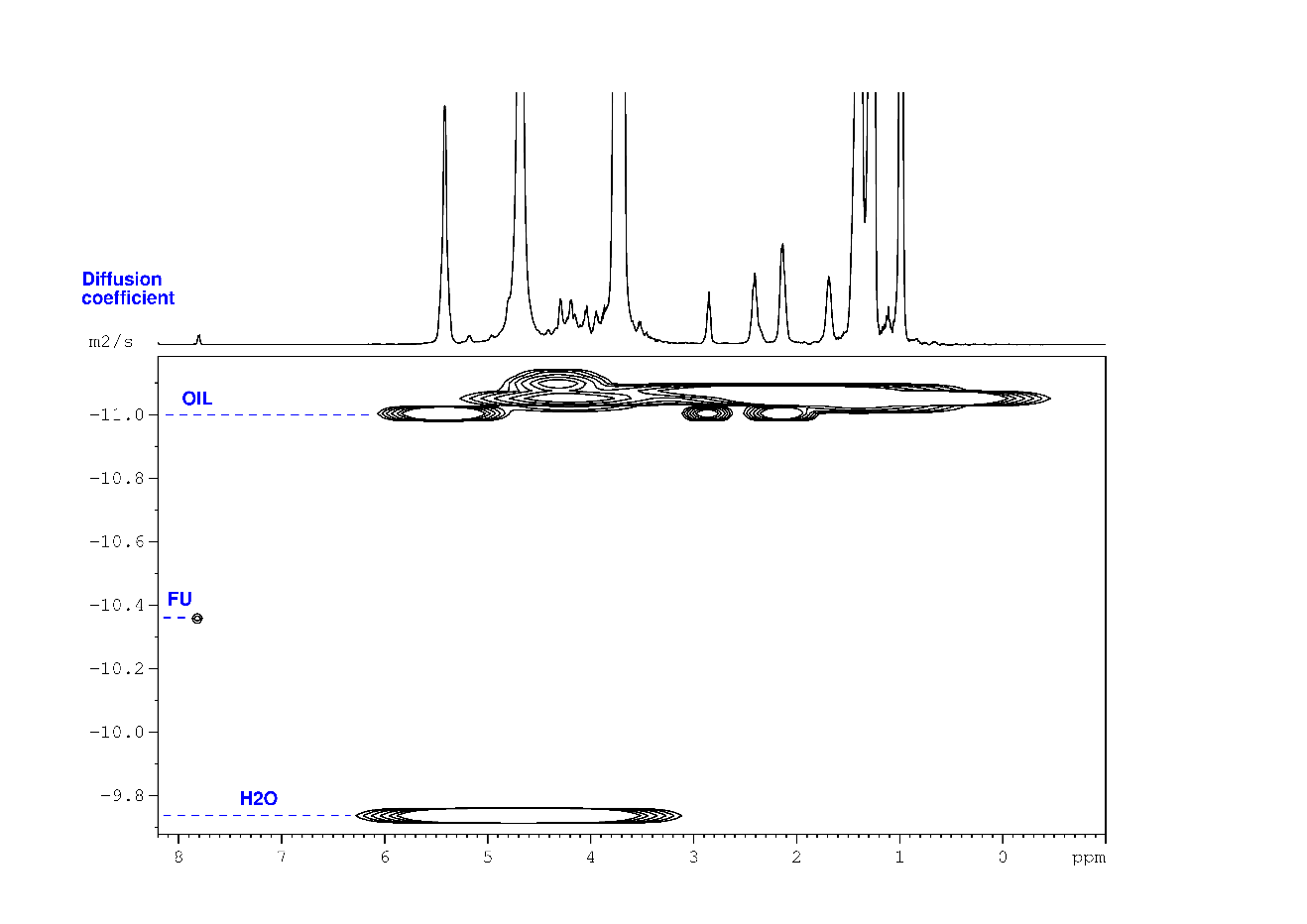


**Figure ESI 7.** **A)** The ^1^H-NMR spectrum of ME_02 that confirms the presence of FU (signal from 7.81 ppm) in the composition and **B)** the DOSY spectrum for the same sample, recorded at 400 MHz.

A)
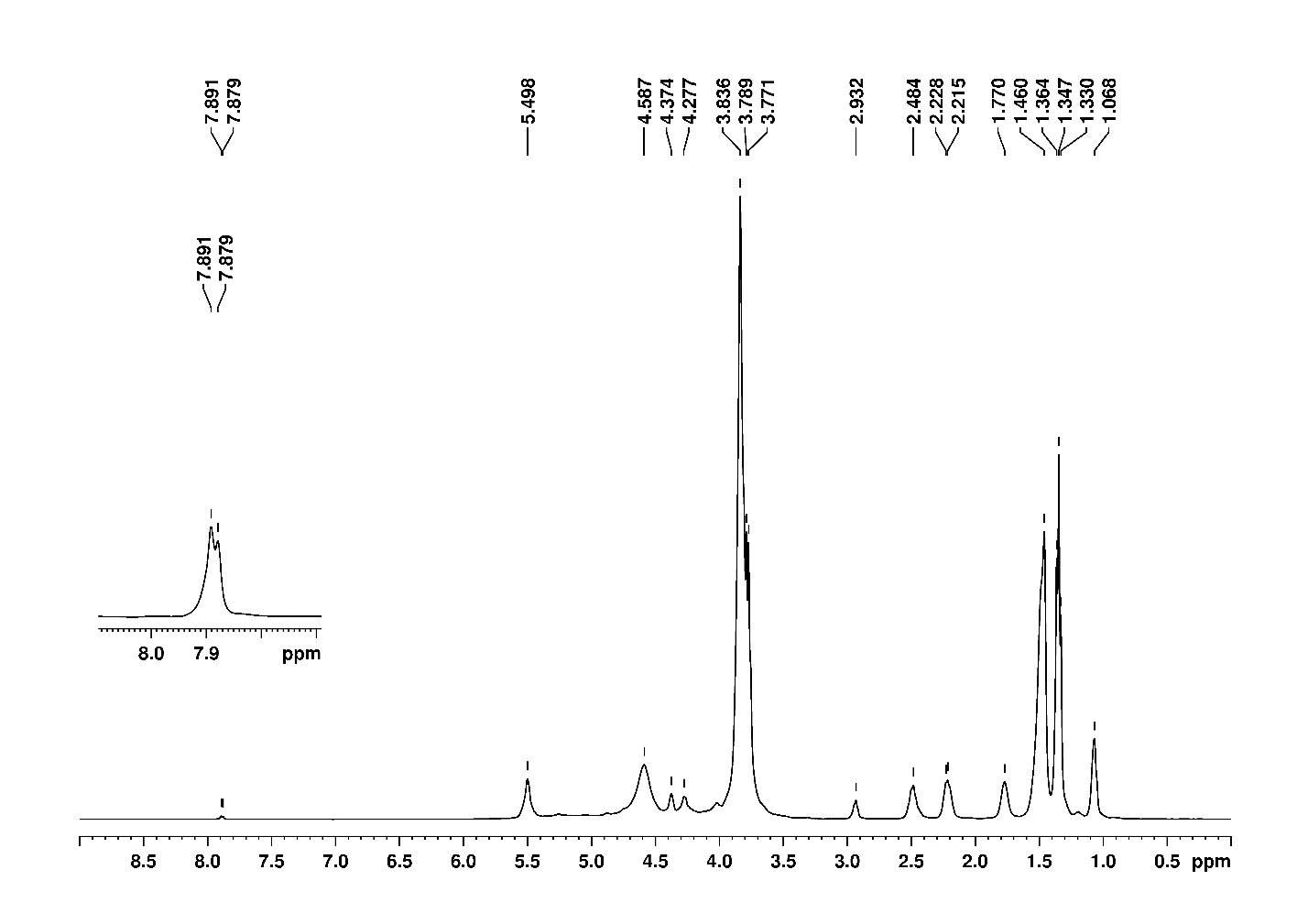


B)
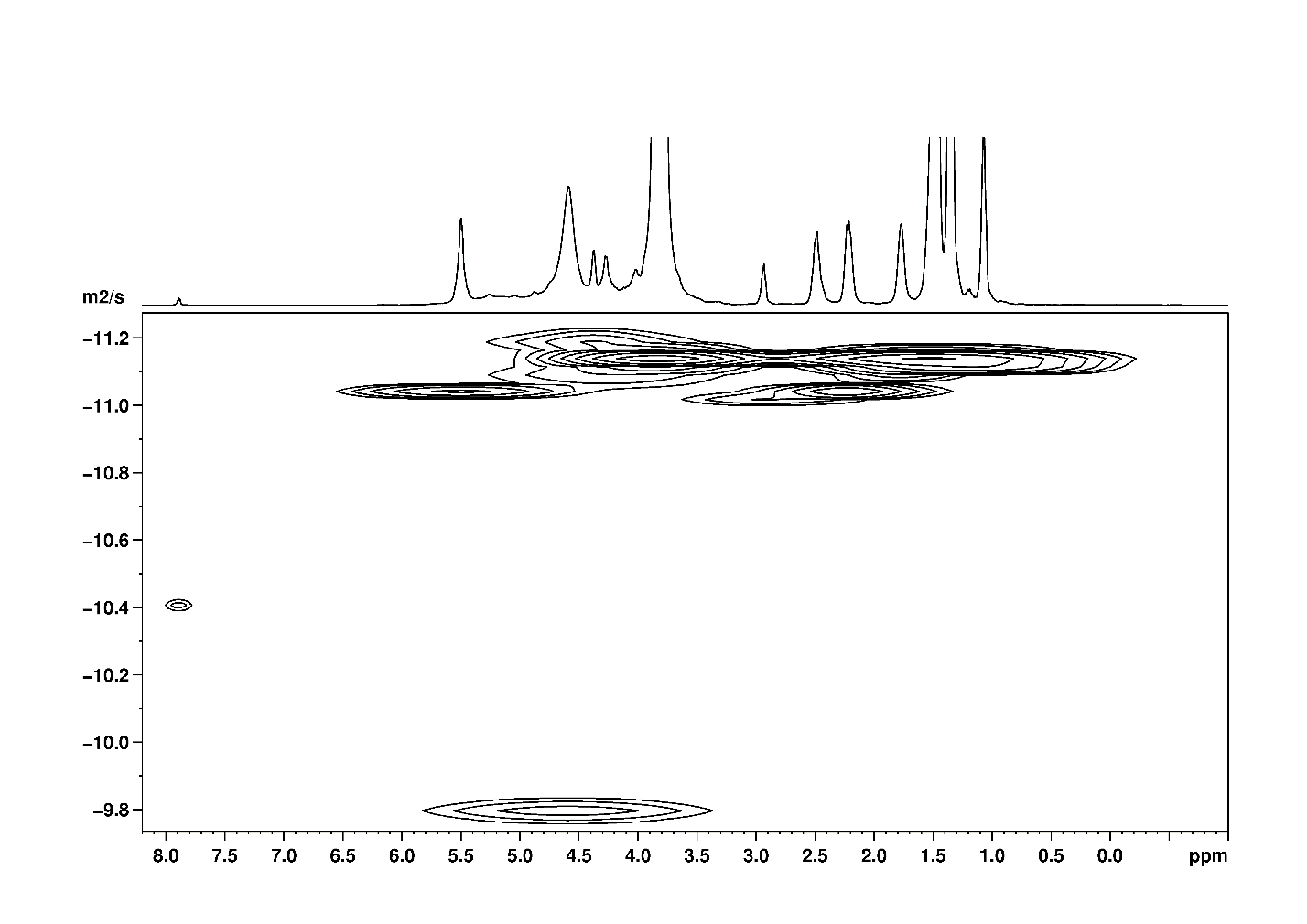


**Figure ESI 8.** **A)** The ^1^H-NMR spectrum of ME_03 that confirms the presence of FU (signal from 7.81 ppm) in the composition and **B)** the DOSY spectrum for the same sample, recorded at 400 MHz.

A)
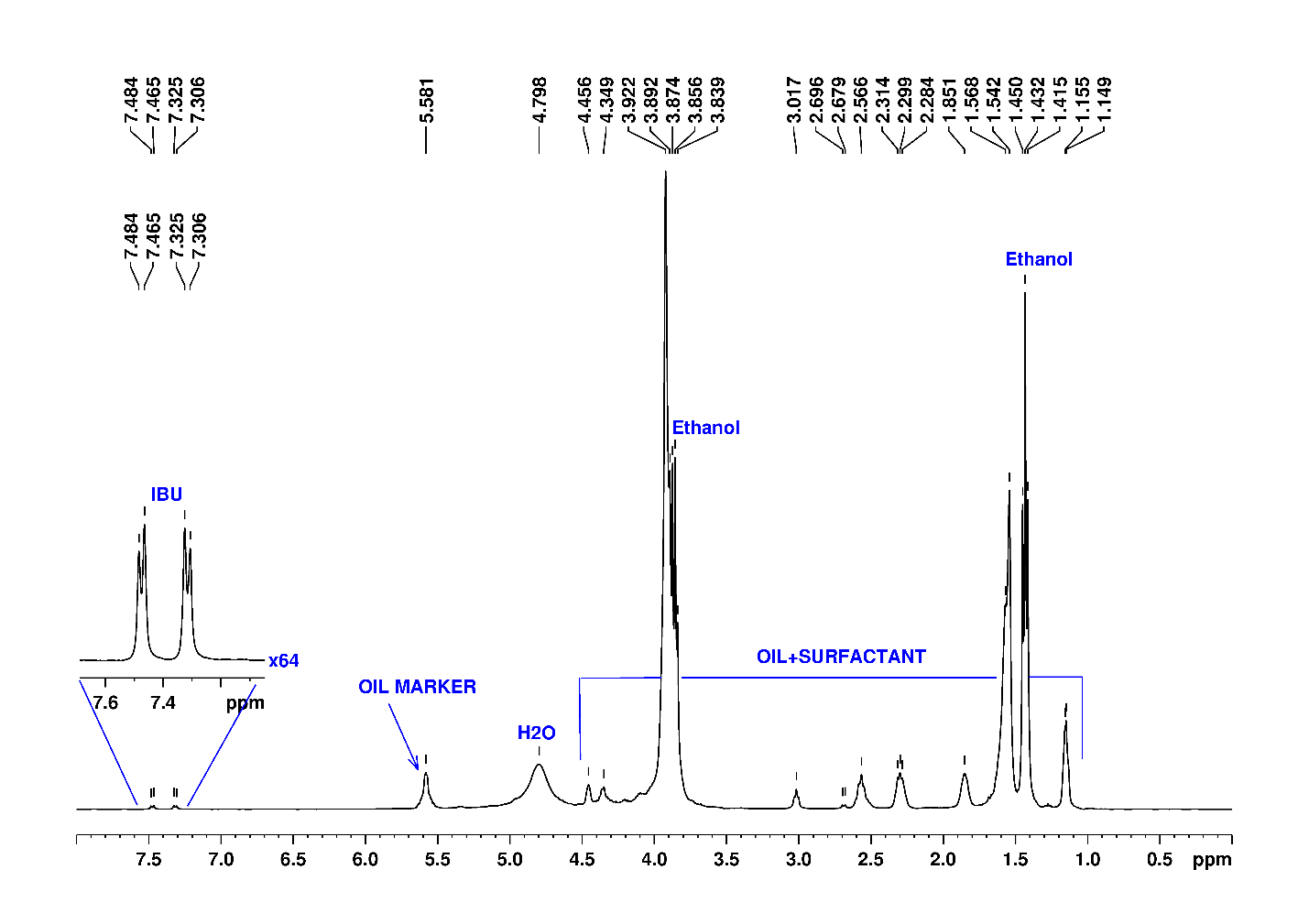


B)
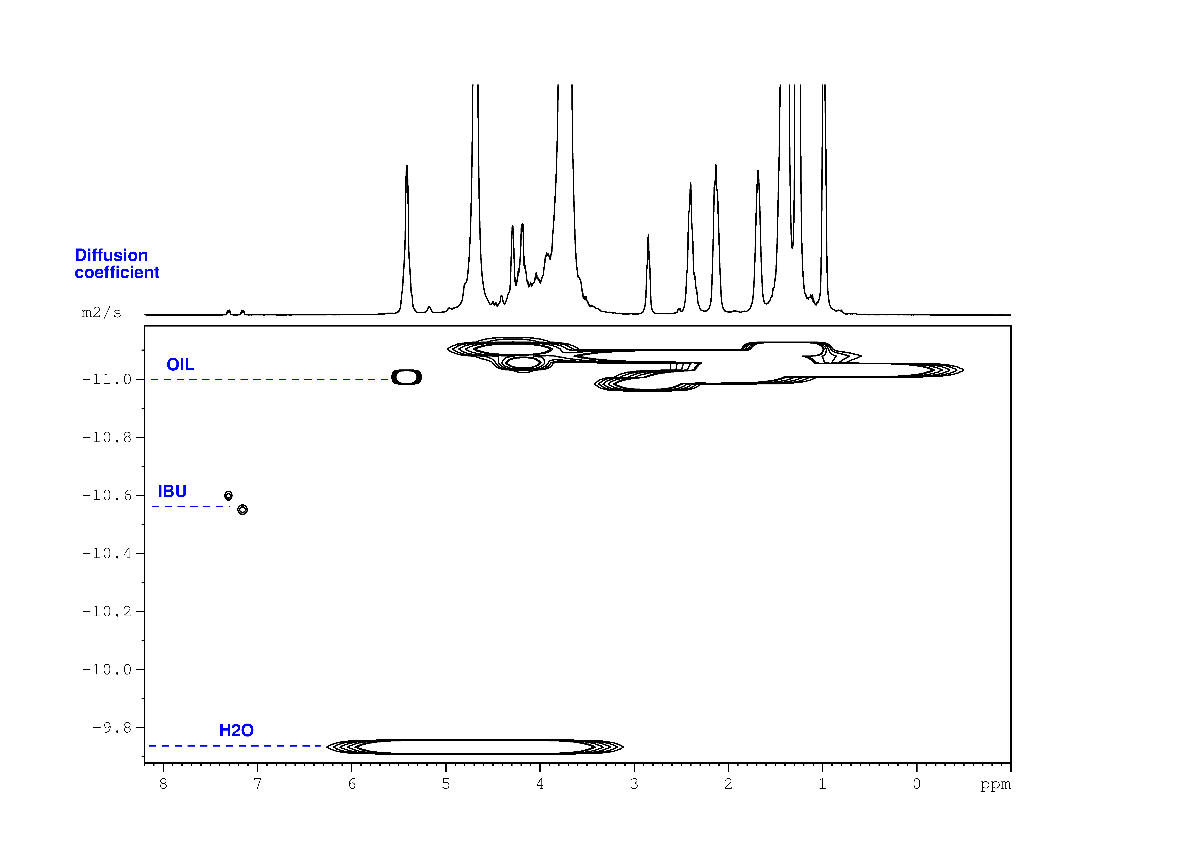


**Figure ESI 9.** **A)** The ^1^H-NMR spectrum of ME_04 that confirms the presence of IBU (signal from 7.31 and 7.47 ppm) in the composition and **B)** the DOSY spectrum for the same sample, recorded at 400 MHz.


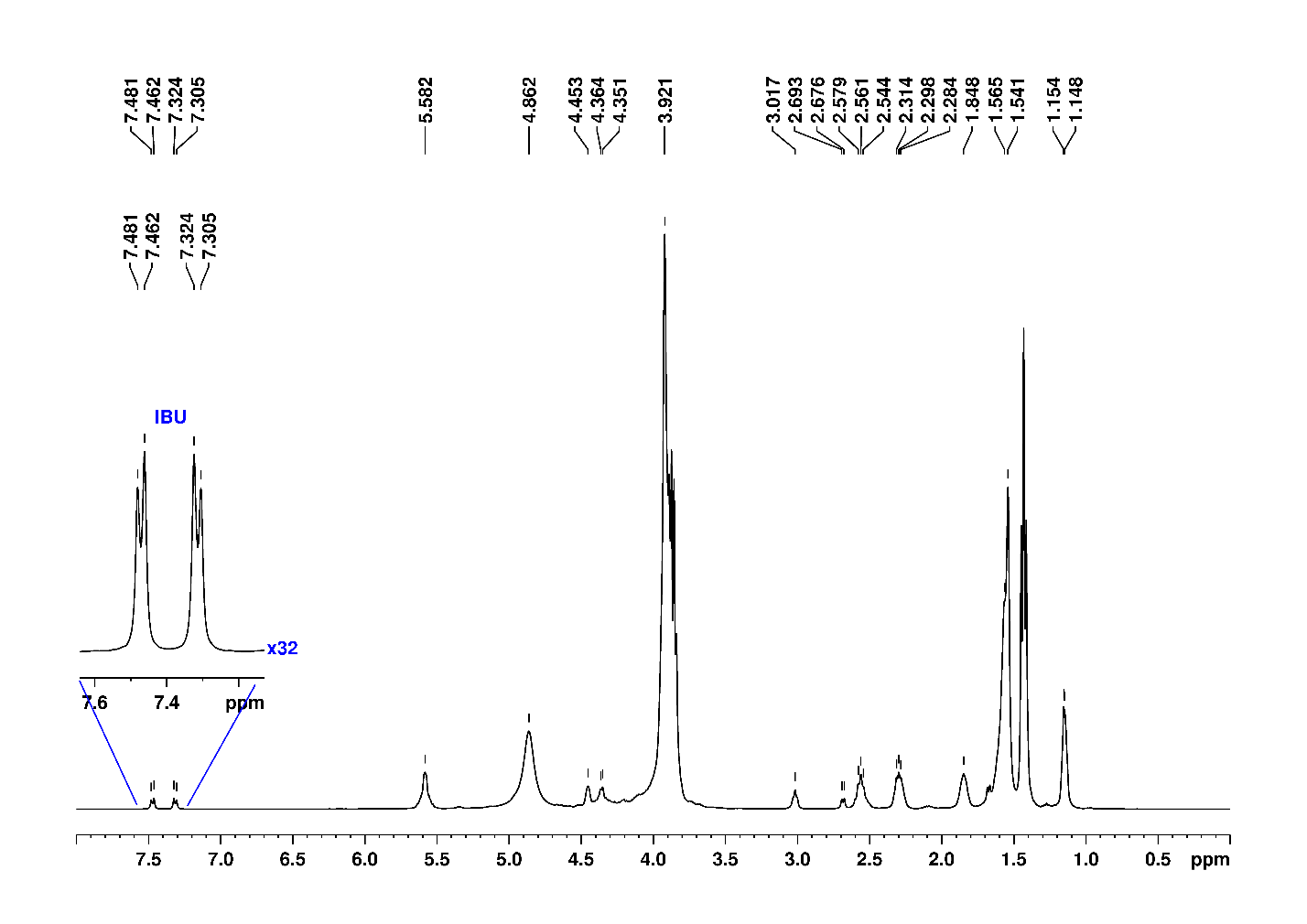


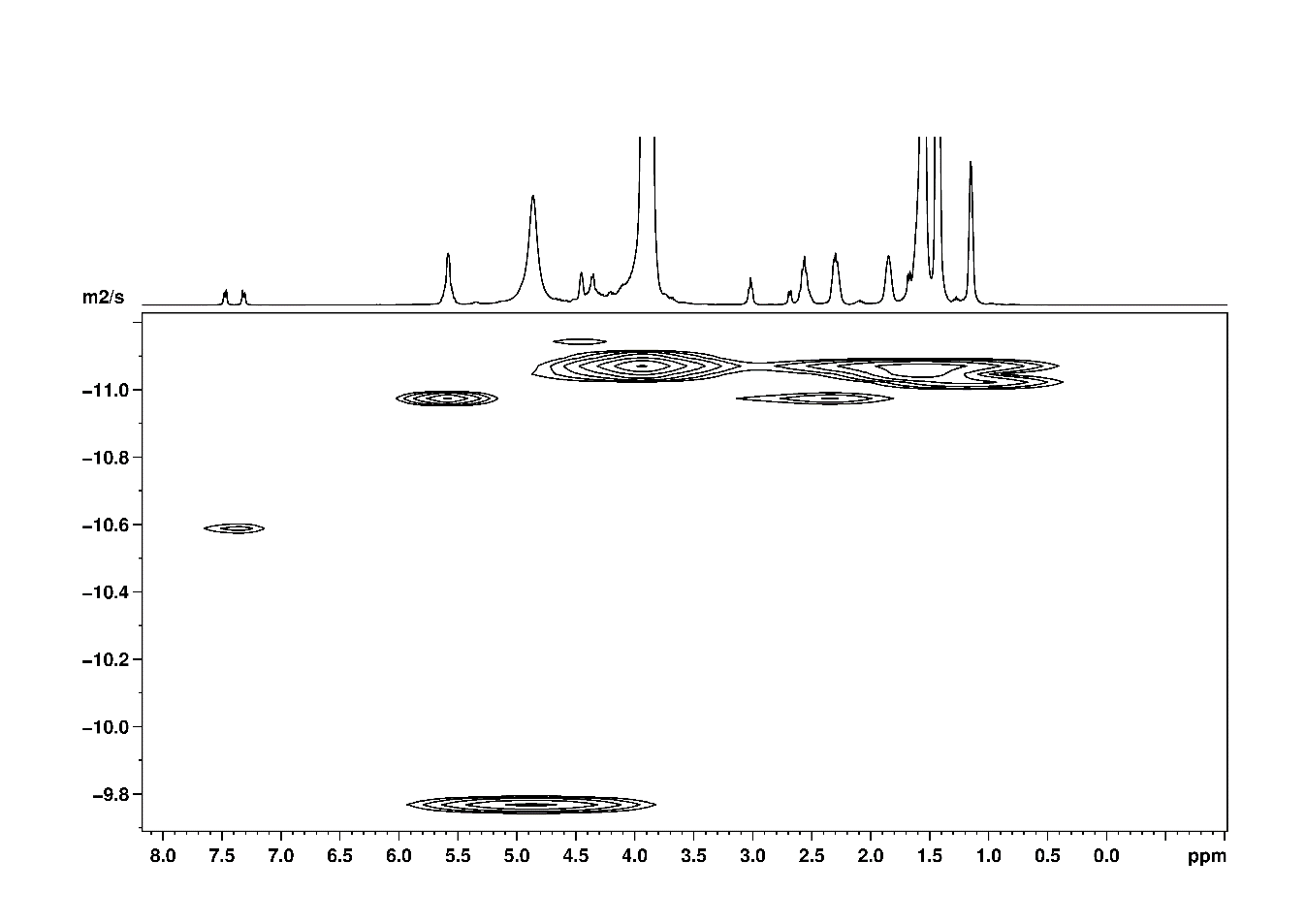


**Figure ESI 10.** **A)** The ^1^H-NMR spectrum of ME_05 that confirms the presence of IBU (signal from 7.31 and 7.47 ppm) in the composition and **B)** the DOSY spectrum for the same sample, recorded at 400 MHz.

A)
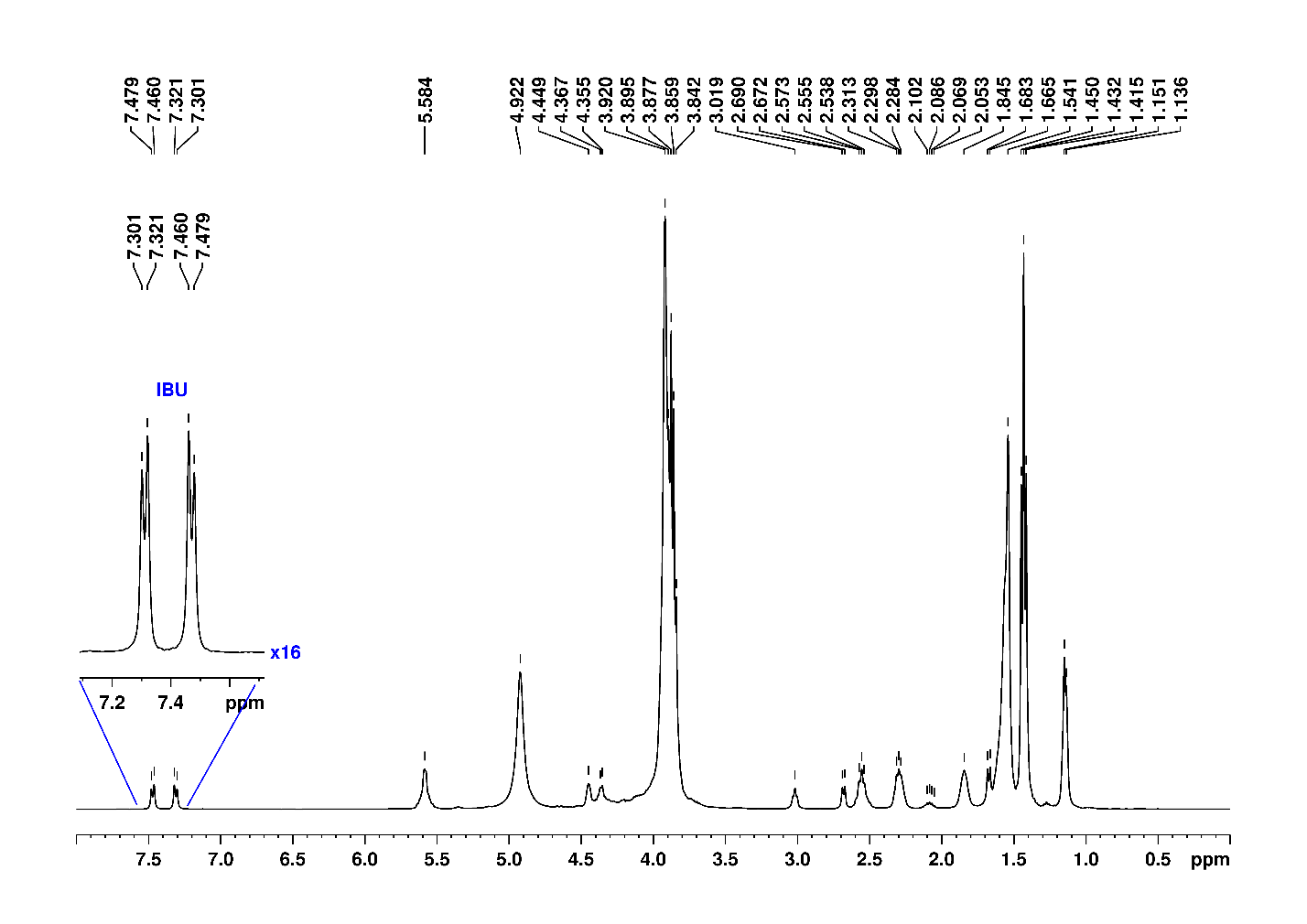


B)
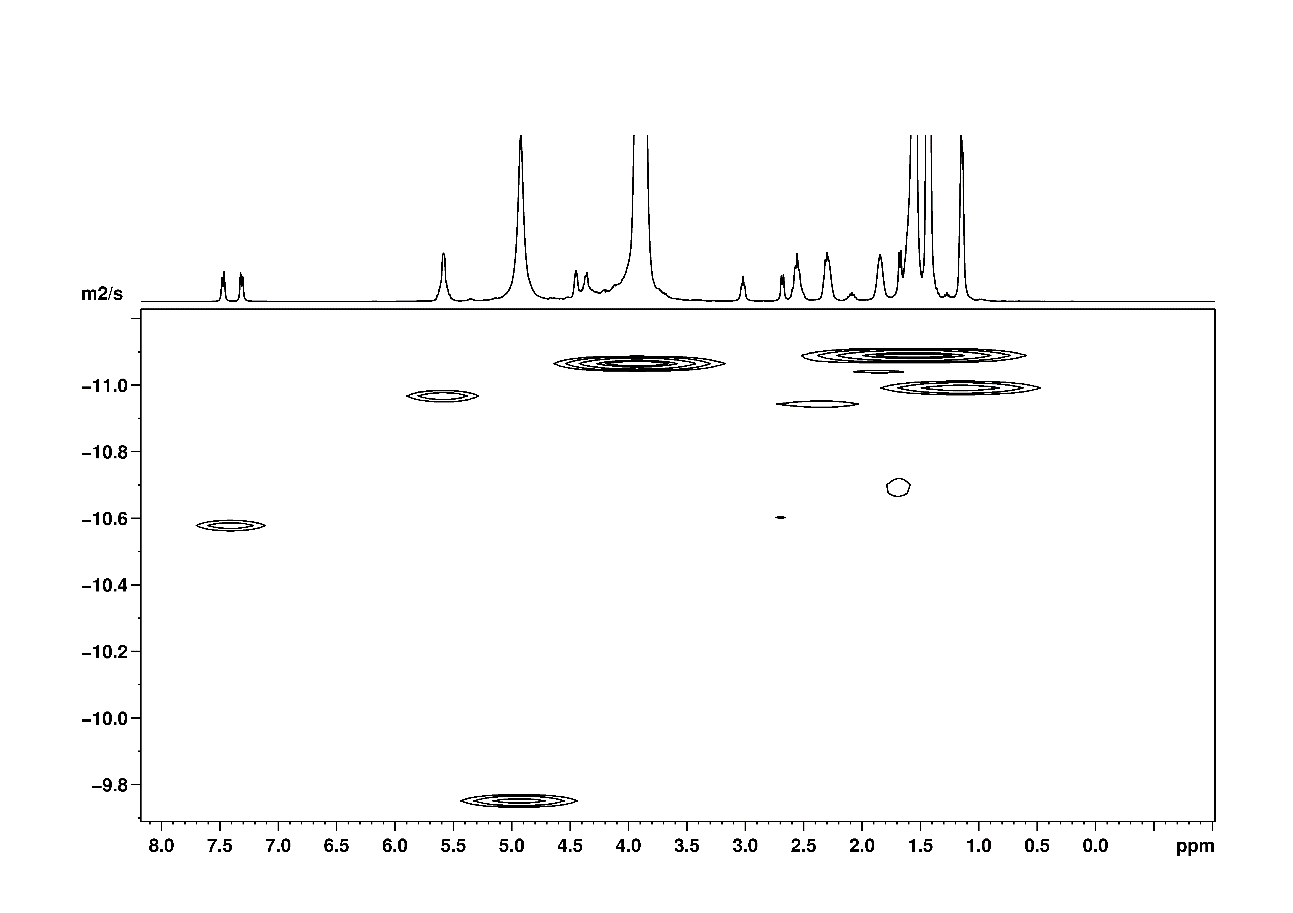


**Figure ESI 11.** **A)** The ^1^H-NMR spectrum of ME_06 that confirms the presence of IBU (signal from 7.31 and 7.47 ppm) in the composition and **B)** the DOSY spectrum for the same sample, recorded at 400 MHz.


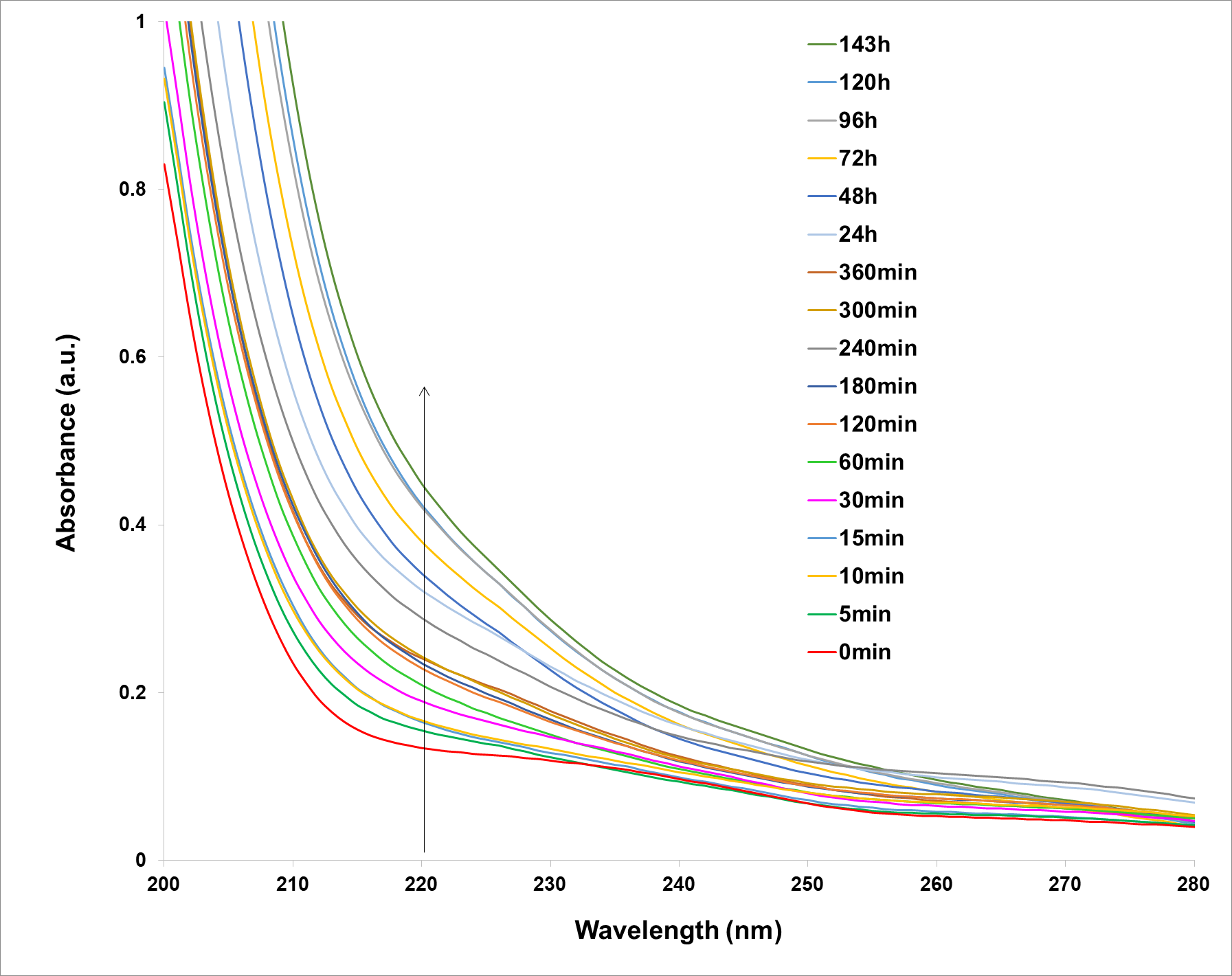


**Figure ESI 12.** UV-Vis spectra of ME after simulating controlled release under the same experimental conditions as for the drug-loaded MEs.


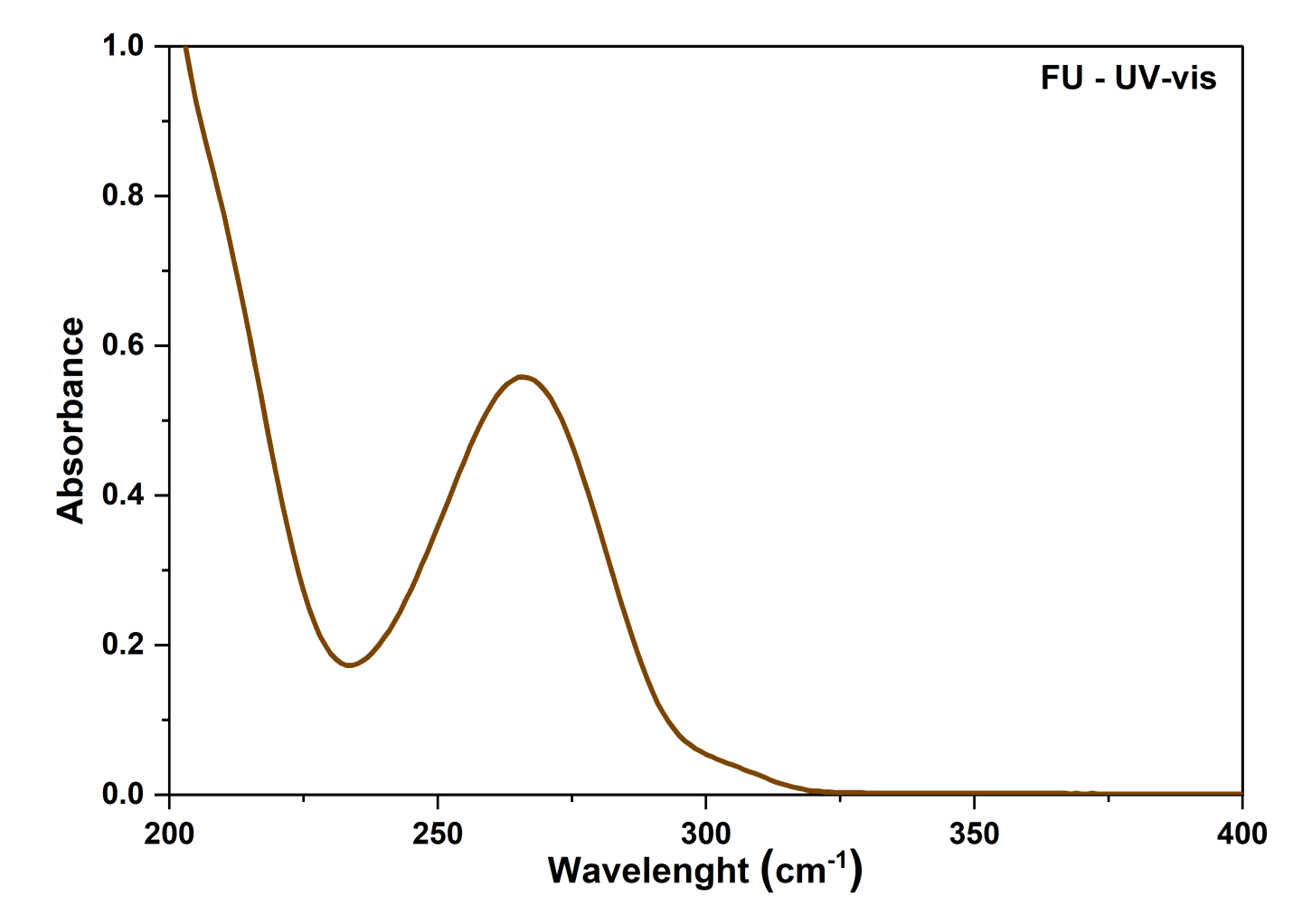


**Figure ESI 13.** UV-Vis spectrum of FU.


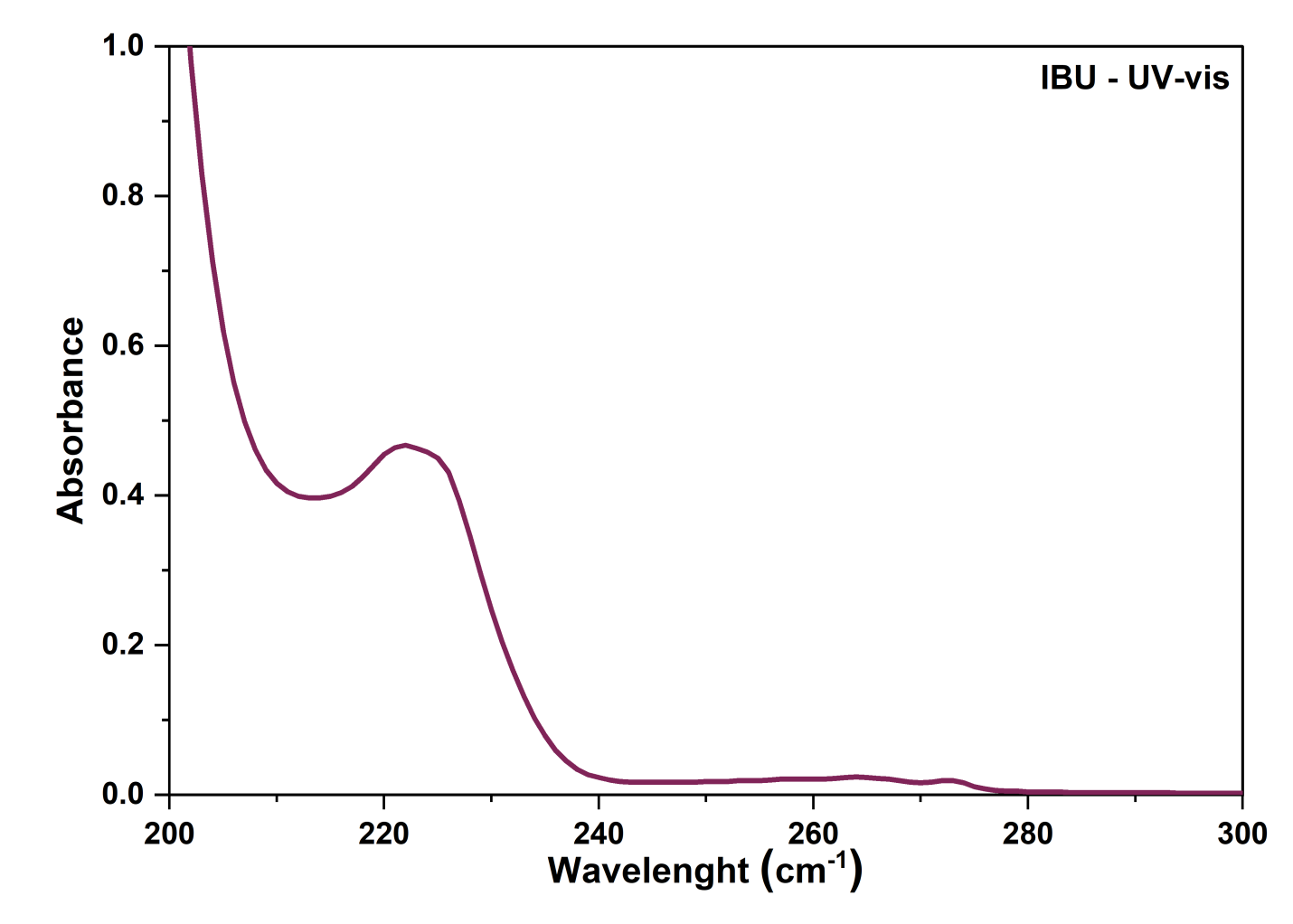


**Figure ESI 14.** UV-Vis spectrum of IBU.

(a)
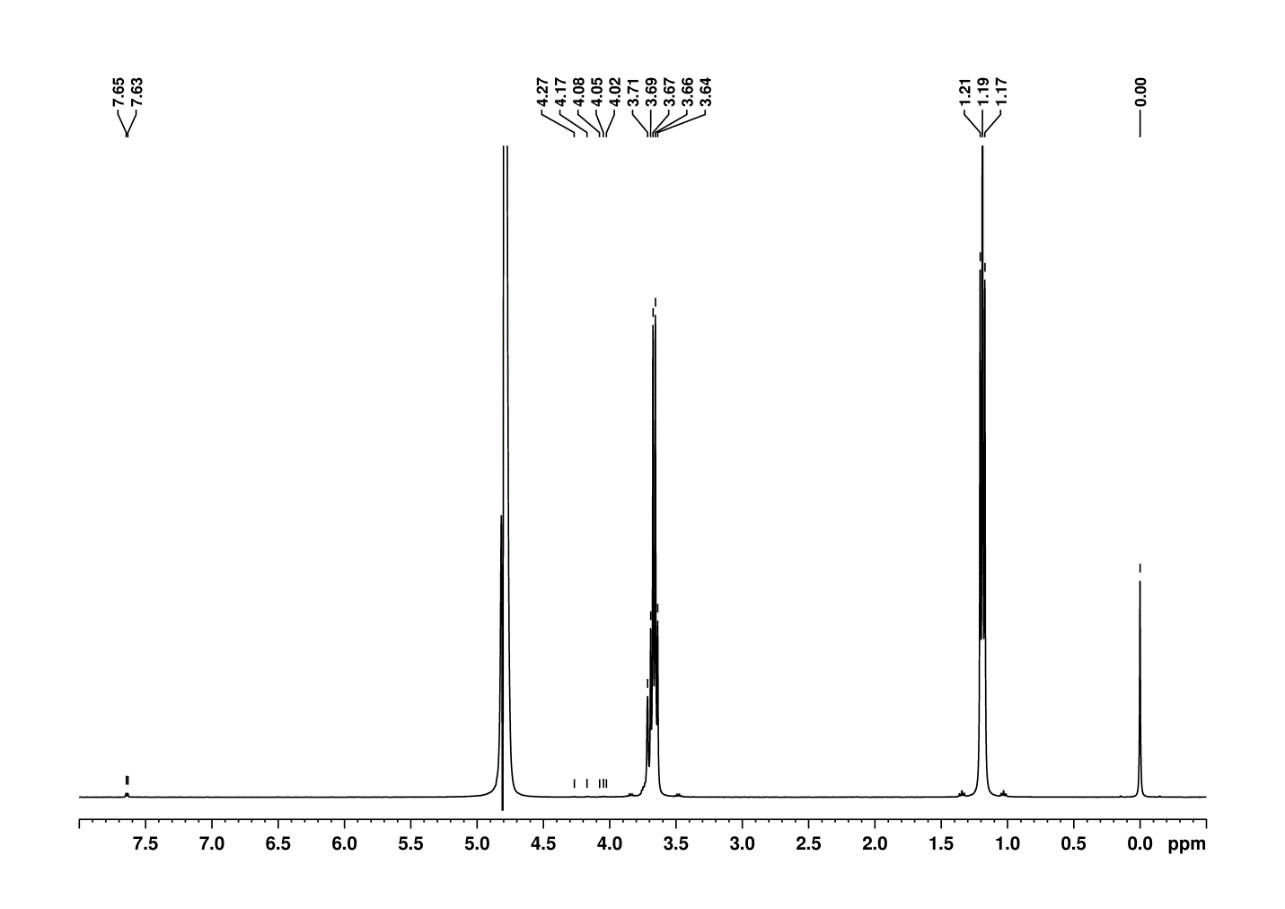


(b)
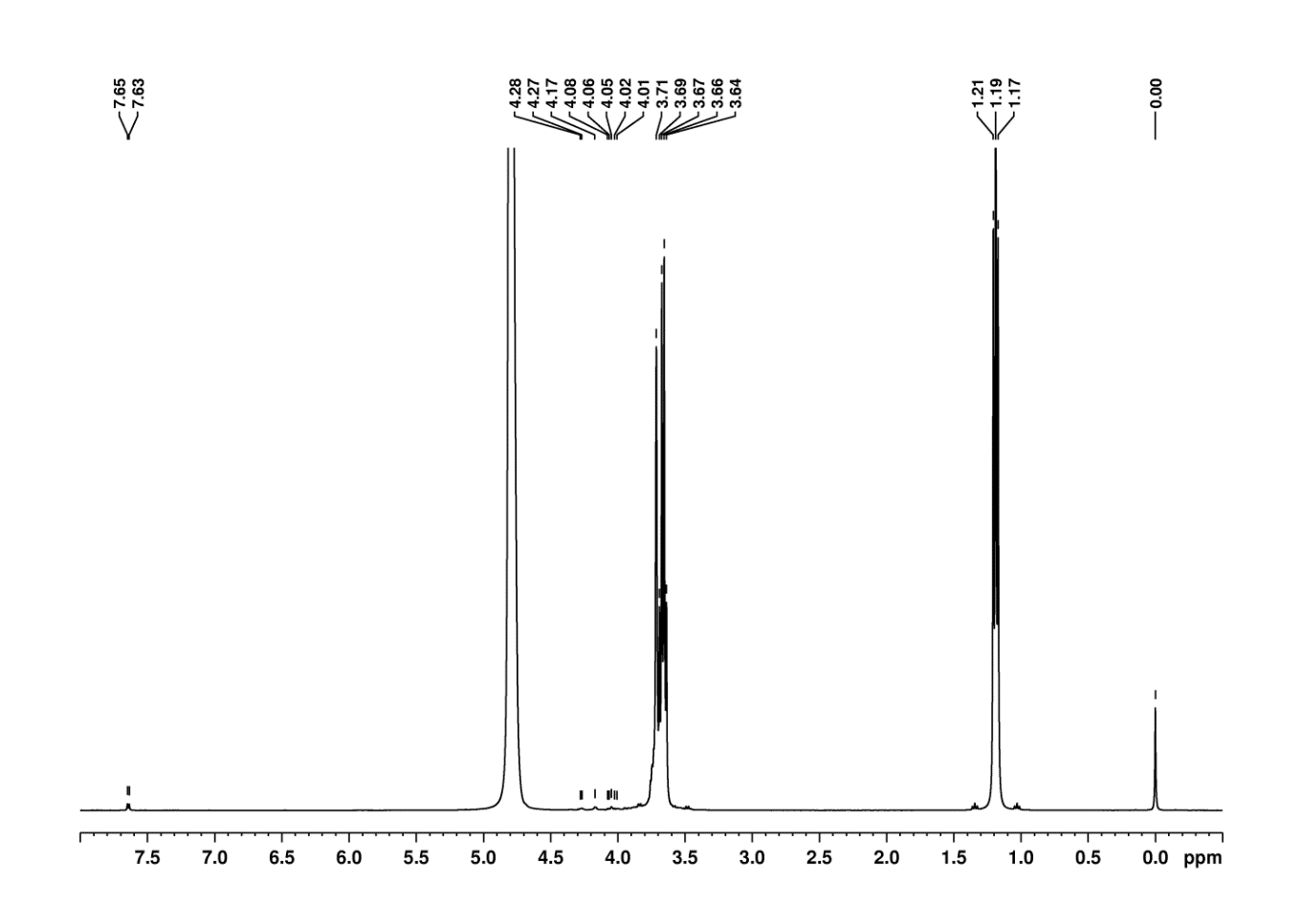


(c)
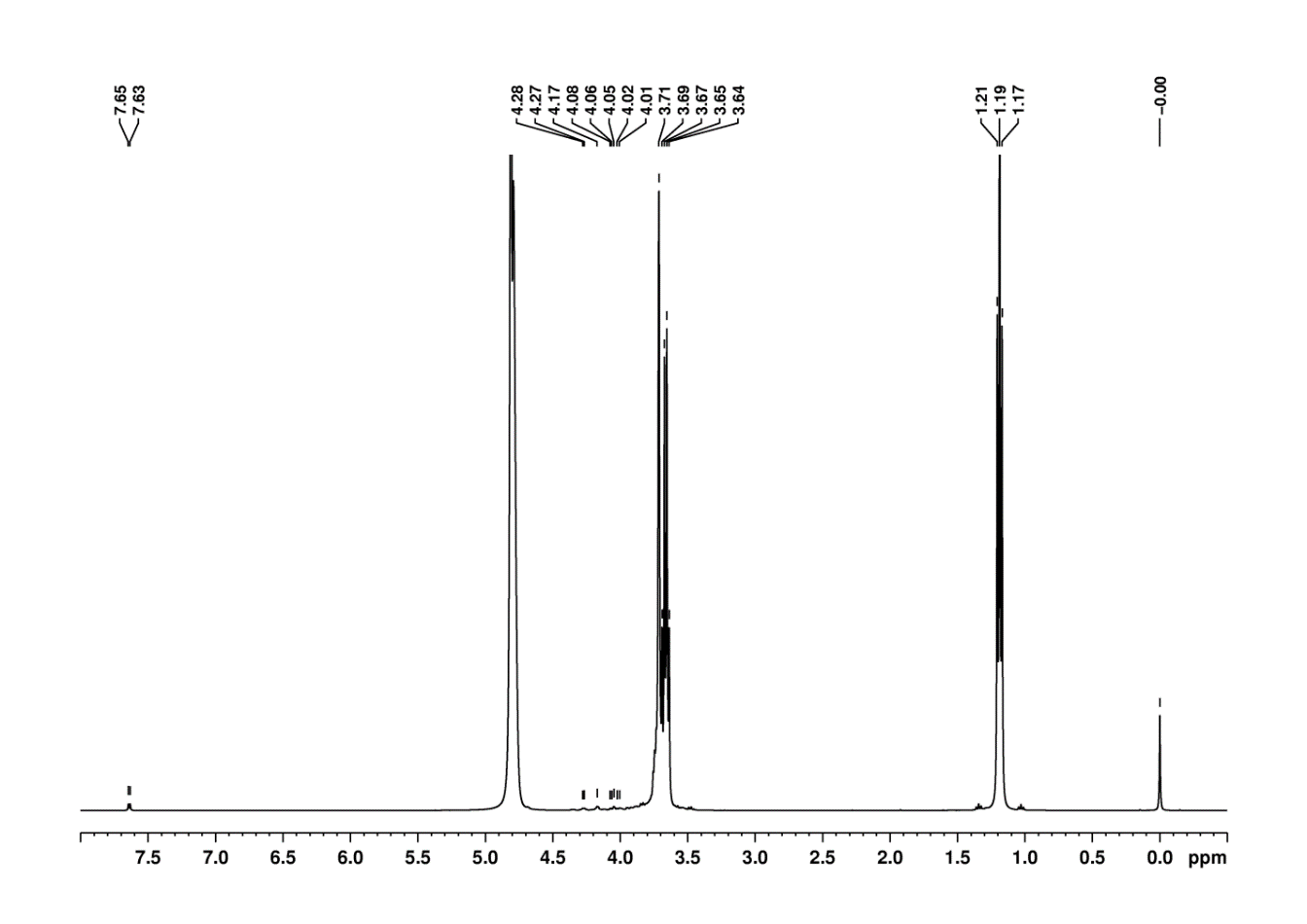


(d)
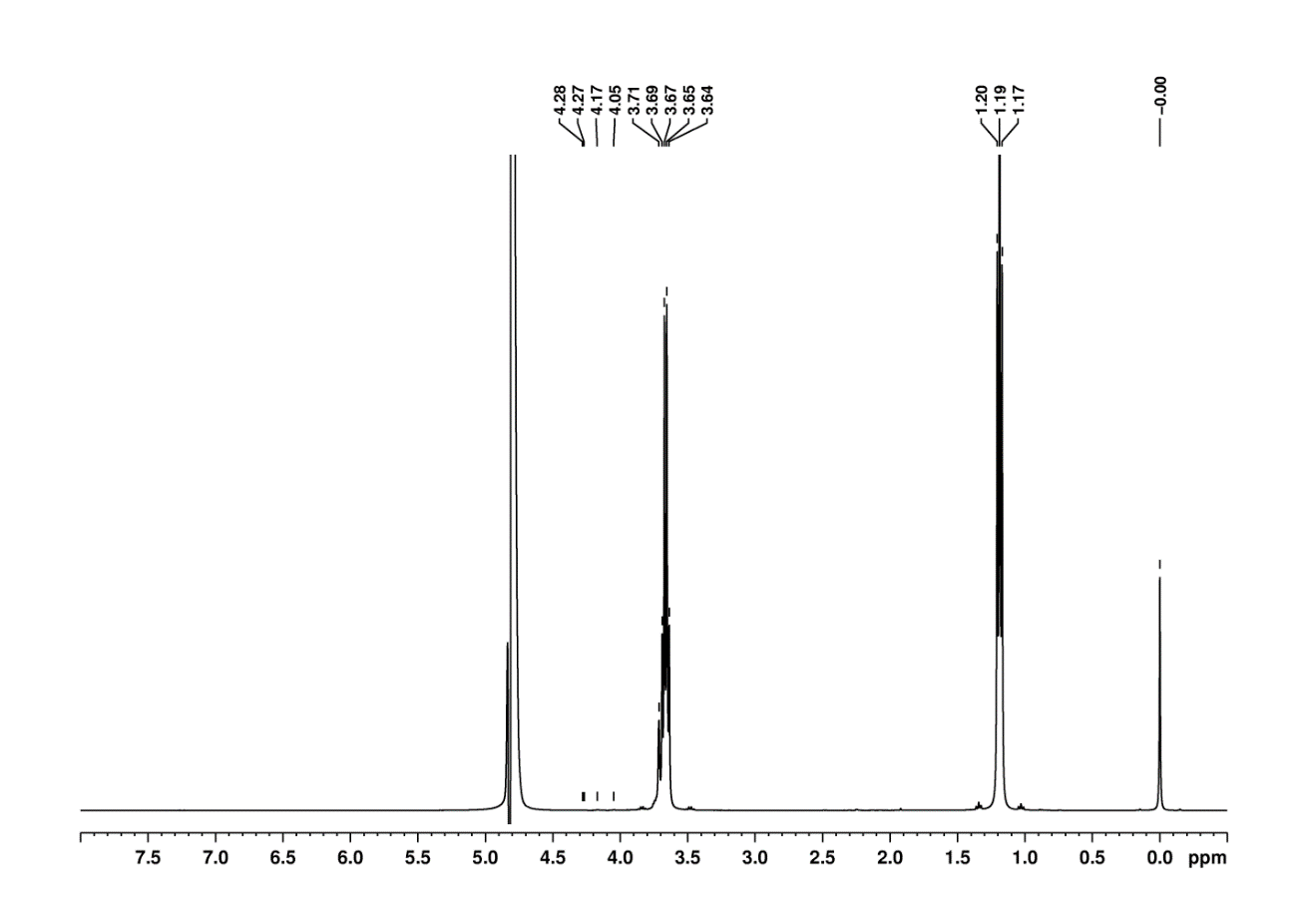


(e)
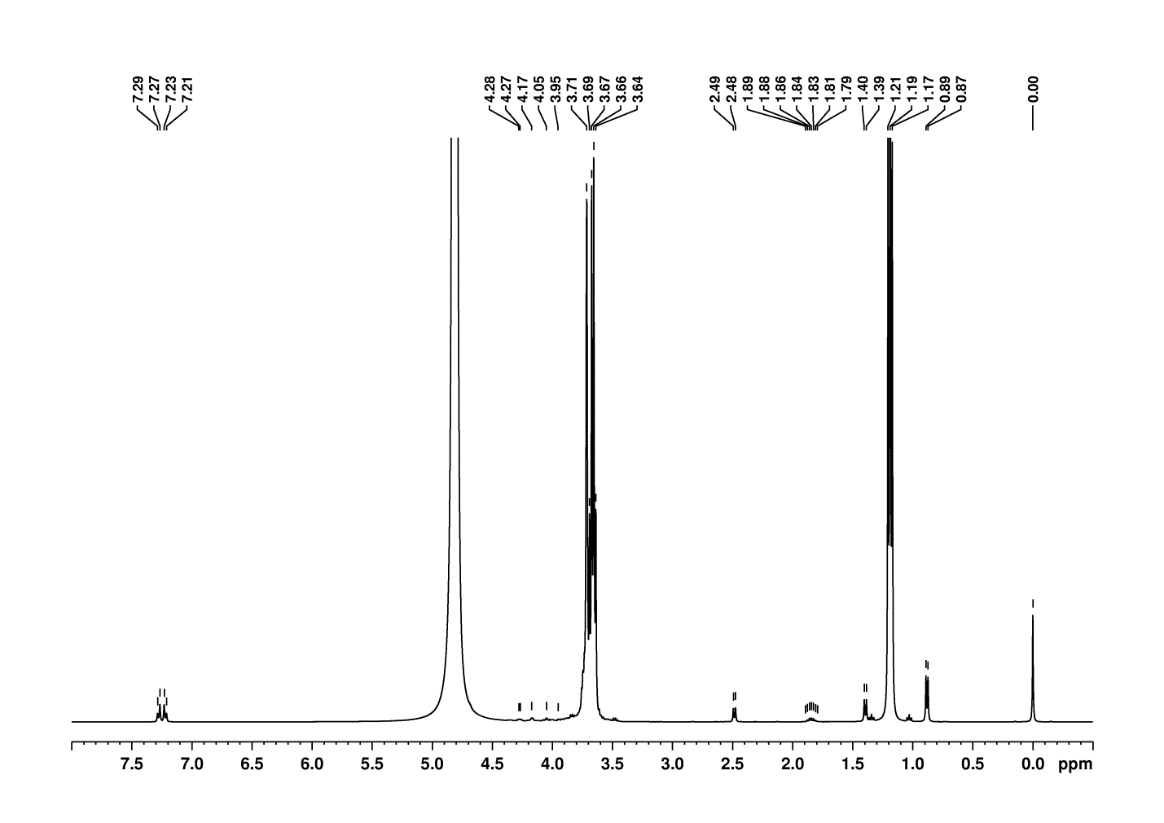


(f)
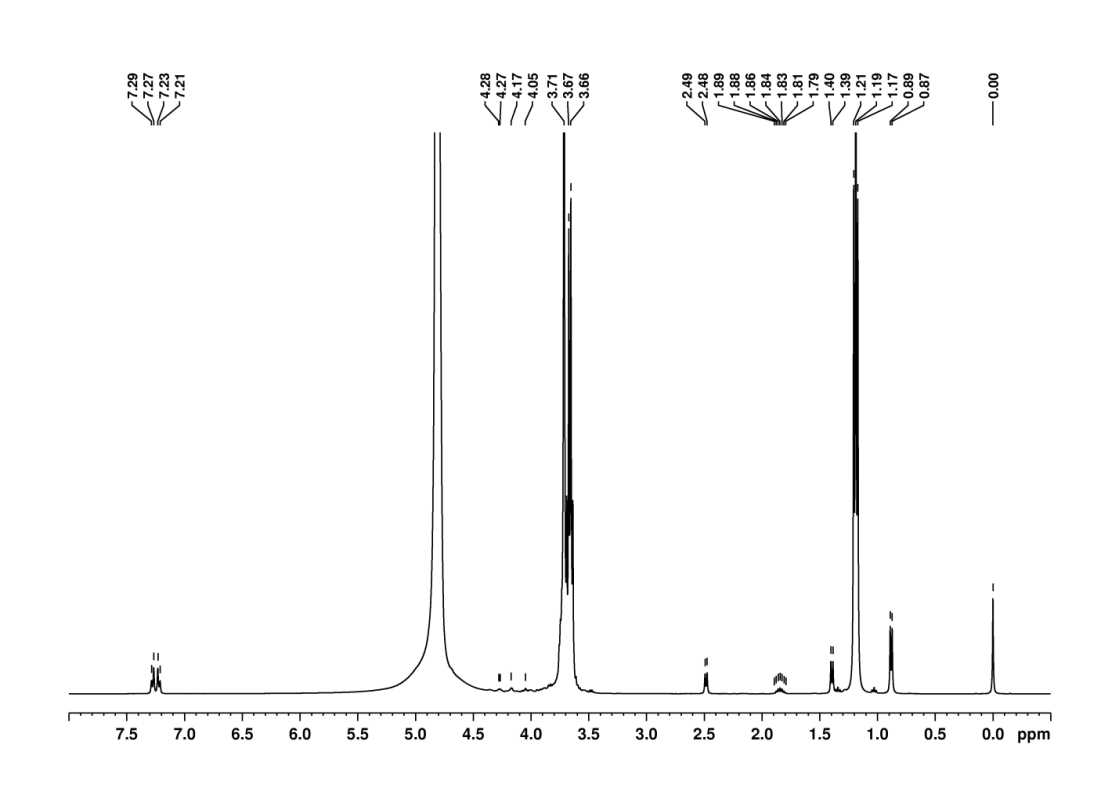


**Figure ESI 15.** ^1^H-NMR spectra of samples after controlled release experiments for 1%FU-3%IBU bicomponent sample: FU (a) after 10 min, (b) FU after 6 h, (c) FU after 24 h, (d) IBU after 10 min, (e) IBU after 24 h, (f) IBU after 96 h.
